# Supplementary material for: Cohort study of a specialist social worker intervention on hospital use for patients at risk of long stay
Source: BMJ Open. 2018 Dec 22;8(12):e023127. doi: 10.1136/bmjopen-2018-023127 (PMC6307584; doi:10.1136/bmjopen-2018-023127)
Supplement: Supplementary data [file bmjopen-2018-023127supp003.pdf]

Supplementary File 3: Detailed description of the specialist social worker-led model of care

An innovative specialist social worker-led model of care was developed and piloted in the study site. This social work led model of care took a holistic approach to the management of complex long stay patient discharges and incorporated: micro, patient and family; meso, organisational; and, macro, external strategies and interventions to manage this patient cohort.

Prior to the implementation of this model of care, individual social workers undertook case management responsibility for complex long stay patients as part of their existing caseload within their allocation ward. These social workers had varying levels of experience ranging from newly graduated to experienced; and carried up to 40 cases at any given point. They constantly juggled the demands of immediate discharges with the more specialised requirements of long stay patients. The social workers had little support or guidance beyond monthly clinical supervision sessions to find appropriate solutions to complex discharge barriers, which inherently impacted length of stay. There was ad hoc early identification with no clear escalation processes. There were little to no relationships with key stakeholders and data collection was rudimentary.

In contrast, the new model of care added an additional level of specialised service delivery which was designed to support and enhance the existing social worker service which continued to undertake 'business as usual', but also expedite discharge through a multi-faceted approach. Underpinned by essential elements including the use of a highly skilled specialist worker, a single point of contact and robust data collection and analysis, the model of care also incorporated social work assistant support to maximise efficiency and align tasks to skill level. Internally, the social work department developed processes to ensure the senior social worker was aware of the progress of each long stay case. A social work clinical case management and reporting database called 'Pathfinder' was developed to allow the senior social worker to track, manage, oversee and provide visibility of all patients on the residential aged care, adults with disability and medico-legal pathways. Ward social workers were required to give regular progress updates to the social work assistant who recorded this information in Pathfinder. Once lodged on Pathfinder the senior social assessed all cases, and tailored specific interventions for each case based on the unique psychosocial barriers to discharge. The system also allowed the senior social worker to monitor, track and oversee all cases, as well as providing regular communication to hospital executive through daily reports to hospital executive and other major stakeholders to ensure the organisation had visibility of the progress of these cases.

Another key element to the model of care was the development of clear and timely internal escalation processes to engage hospital executive in decision. Protocols for these interventions were formalised and their benefits were promoted to hospital management and staff to ensure they became embedded into clinicians' everyday practice. The senior social worker, in consultation with the Director of social work escalated stalled cases or 'stranded patients' that posed a significant discharge delay or reputational risk to the organisation due to external institutional delays, family refusal of care, or legal issues.

Externally the model of care implemented pathways and partnerships with key agencies and care providers to ensure timely and appropriate discharges. A nursing home vacancy register was developed alongside a targeted strategy to develop key relationships which resulted in priority notification of vacancies and improved outcomes for patients, especially those with challenging behaviours who previous were difficult to place. Relationships and pathways were also developed with key government agencies, improving their understanding of hospital discharge timeframes and ultimately leading to more responsive turnaround on decisions which had previously taken weeks or months.

An important element of this model was the inclusion of a social work assistant who successfully assisted both the senior social worker and also the ward social workers with sourcing nursing home vacancies using the nursing home vacancy register as well as through the relationships developed with facilities. The role also assisted with time consuming administrative tasks particularly for residential aged care placements such as emails, phone calls, paperwork collation and the maintenance of Pathfinder, nursing home vacancy and other databases. This support was essential to the success of the model by allowing the senior social worker the time to provide the necessary consultations and specialist advice.

Lastly, the model of care also drew on the social workers' specialist skills and knowledge to undertake an advisory role thereby influencing both the organisation and external networks through membership on key committees. The role has been instrumental in the development of new policies and services that ultimately improve outcomes for complex long stay patients. An example of this was the development of strategies for advocating for patients' rights in the face of external institutions' abrogation of their responsibilities to patients such as refusal of ongoing care.

Although developed in a large urban hospital, the model has potential to be modified and scaled to meet local needs and can be implemented without the assistance of a support worker.
