# Supplementary material for: Cohort study of a specialist social worker intervention on hospital use for patients at risk of long stay
Source: BMJ Open. 2018 Dec 22;8(12):e023127. doi: 10.1136/bmjopen-2018-023127 (PMC6307584; doi:10.1136/bmjopen-2018-023127)
Supplement: Supplementary data [file bmjopen-2018-023127supp001.pdf]

Supplementary File 1.

Psychosocial Barriers to Discharge Identified from Audit of Long Stay Patients

Barriers to discharge	Examples and/or impact on patient, carer, family
Family complexity (dysfunction or conflict)	Family members may disagree on whether patient should go into residential aged care or go home or disagreeing on care arrangements for a patient post-discharge. This could also include family estrangement, inability to locate family, or situation where the partner of a patient does not want the patient discharged because the partner is currently involved in another relationship.
Domestic and family violence/Elder abuse	This could be suspected or confirmed. It is often difficult to confirm elder abuse, especially if the patient has cognitive impairment, dementia, mental health issues, etc. However, even if abuse cannot be confirmed, the health care professional cannot discount the risk to the patient if he/she is discharged into the care of a suspected abuser.
Substitute decision making	The patient may be awaiting assessment by a civil or administrative tribunal, which is an independent, accessible tribunal that efficiently resolves disputes between parties on a range of matters, such as establishing who is the substitute decision maker if there are complex family dynamics (for example, the patient recently formed a new partnership but has adult children), or the patient has become incapacitated or unable to make sound decisions on their behalf.
Carer stress	Carers indicate that they can no longer manage the demands of caring for the patient and/or are experiencing burnout.
Substance abuse	Patient substance abuse may reduce accommodation options. Substance abuse may also manifest in patient aggression.
Homelessness	For homeless patients, there are usually no viable discharge options.
Challenging behaviours	This is usually in reference to challenging behaviours displayed by the patient, for example, physically or verbally abusive behaviour towards others; not complying with medical care; sexually inappropriate behaviour; dementia related behaviours (e.g. wandering); verbal aggression. These types of behaviours make it harder to find facilities who will accept patients who are likely to impact on the quality of life of other residents or upset staff.
Disability	This usually refers to patients with disabilities where finding suitable out of hospital placements or in-home carers may be difficult.
Financial stress	This could be in relation to the patient and/or the family and could manifest as significant debt or lack of regular income; whereby, patient may not be able to afford residential aged care.
Residential Aged Care	A situation where the patient requires residential care but there is no suitable vacancy or alternatively, the patient may simply be awaiting placement.
Disability Care Planning	A patient may need services at home and be awaiting services at home to be put in place; or there is a lack of suitable accommodation options to meet the disability needs of the patient.
