# Supplementary material for: Cohort study of a specialist social worker intervention on hospital use for patients at risk of long stay
Source: BMJ Open. 2018 Dec 22;8(12):e023127. doi: 10.1136/bmjopen-2018-023127 (PMC6307584; doi:10.1136/bmjopen-2018-023127)
Supplement: Supplementary data [file bmjopen-2018-023127supp002.pdf]

Supplementary File 2. Conceptual Case Study Exemplars[1] of Long Stay Patients with Complex Psychosocial Barriers to Discharge

Case 1 Jack

Jack is an Aboriginal man in his mid-sixties who **at the time of admission had been homeless for several years.** He had previously resided in supported accommodation, but had been evicted for abusive behaviour. The patient had no family or support network. He had a history of frequent admissions and on one occasion had spent four months in hospital. In the month prior to the specialist social worker's intervention, he was admitted to hospital on three occasions via the Department of Emergency Medicine (DEM). Typically, he would be brought to DEM by the police after being found semi-conscious lying on the ground in a public space. Alternatively, a local homelessness service would organise his transfer to hospital.

Jack has diabetes and is incontinent. He posed many challenges to nursing and allied health staff while in hospital because of his abusive and erratic behaviour. Substitute decision makers were involved because of his cognitive impairments. At the time of his latest admission, DEM staff facilitated a clinical case consultation and he was identified for follow up by the specialist social worker. **Early identification of this potential long stay patient resulted in the specialist social worker calling a stakeholder meeting with executives in DEM and a medical consultant to plan his management, including developing a behavioural management plan**. Identified psychosocial barriers to discharge included homelessness, substitute decision making, lack of family support, challenging behaviours, resistance to being discharged, and finding a suitable residential care facility.

Jack spent a total of ten days in hospital on this occasion. The specialist social worker intervened on day nine and, and through intensive case management, was able to negotiate a nursing home placement in a nearby coastal town. Although he was initially uncooperative, the specialist social worker worked with the ward social workers to identify the source of his resistance to discharge. The patient was counselled and encouraged to express his fears about discharge. The specialist social worker used a directive style of communication and negotiated a workable discharge plan for the patient that involved procuring a wheelchair for his use after discharge. He was transferred to the nursing home the next day. While the patient did move into the nursing home, he was later admitted to the local hospital and then self-discharged against medical advice. He subsequently hitchhiked back to the metropolitan hospital where he had been a frequent presenter. However, since that time he has not presented again.

Case 2 - Ben

Ben is a divorced male in his late eighties who had been residing at home at the time of admission. He was from a Filipino background, fluent in English, and had **no family support in Australia.** In the previous year, Ben had five hospital admissions over a nine month period. During the latest admission due to multiple medical conditions and his inability to cope at home, he was admitted to the Geriatric Evaluation and Management (GEM) unit for rehabilitation. While Ben valued his independence, his physical impairments and social isolation put him at risk of further injury. He was assessed as needing residential care because of his inability to carry out activities of daily living.

---

[1] Note: These case studies are conceptual composite cases highlighting a range of psychosocial barriers to discharge for long stay patients or patients at risk of long stay in hospital. No one case represents a single patient.

Ben presented as angry and resistant to staff's attempts at discharge planning. Although he had signed a residential care agreement, he subsequently displayed an unwillingness to follow through with the residential care pathway. **Despite the ward social worker's attempts to facilitate a placement, three months had elapsed with no progress.** The specialist social worker re-assessed the case and identified the barriers to discharge including a lack of family and social support, a strong resistance to considering residential care options, and financial stress. The specialist social worker worked with the ward social worker and the GEM unit manager to devise a systematic step-by-step case management plan, which included escalating the case to the executive because of the stalemate surrounding the patient's discharge. With executive support, the patient subsequently agreed to enter an interim care facility after his options were put to him in a formal letter. He was re-admitted to hospital after two weeks in interim care, but was later discharged to a nursing home which he attended voluntarily. **Ben's total length of stay was 101 days.**

Case 3 – Mark

Mark is a male in his late seventies and resides in a nursing home. He has a range of cognitive impairments. He was transported to the Department of Emergency Medicine (DEM) at the request of the nursing home. **Despite being assessed as discharge-ready, the nursing home refused to take him back to the facility.** Mark exhibited challenging behaviours including verbally abusive behaviour and inappropriate touching of staff and patients. At admission, the specialist social worker assessed the patient as being at risk of prolonged hospitalisation due to his behavioural issues and the nursing home's failure to comply with its responsibilities. **Although the ward social workers had been advocating for his return to the nursing home, this was met with strong resistance.** As a result of the staff's inability to convince the nursing home to readmit the patient, the specialist social worker became involved with the case two days post-admission. She consulted with the Director of Social Work about strategies to manage the case and subsequently escalated the matter to the hospital executive. The hospital executive, acting on the specialist social worker's advice, reminded the manager that the patient had security of tenure and quoted relevant sections of the Aged Care Act to advocate for his return. The stalemate around discharge was subsequently resolved at the executive level of the hospital and nursing home following a stakeholder meeting initiated by the specialist social worker. **Mark was discharged after six days in hospital.**

Case 4 – Gerry

Gerry is a divorced male in his mid eighties. Prior to his hospital admission he lived independently in his own unit. Gerry was admitted to an acute medical ward after a fall. After 17 days, his condition was classified as non-acute but he subsequently acquired an antibiotic resistant infection and was admitted to the infectious diseases ward. The patient was assessed as needing interim care pending his return home or entering a residential facility. However, his infectious status represented a significant discharge barrier and he was declined admission by one interim care facility. **Forty days post-admission, the specialist social worker intervened.** Through her previous work in establishing a strong working relationship with the interim care facilities in the local area, the specialist social worker was able to advocate for a private room for the patient. **He was discharged from hospital the next day.**
