# Supplementary material for: Cohort study of a specialist social worker intervention on hospital use for patients at risk of long stay
Source: BMJ Open. 2018 Dec 22;8(12):e023127. doi: 10.1136/bmjopen-2018-023127 (PMC6307584; doi:10.1136/bmjopen-2018-023127)
Supplement: Author's manuscript [file bmjopen-2018-023127.draft_revisions.pdf]

BMJ Open

BMJ Open is committed to open peer review. As part of this commitment we make the peer review history of every article we publish publicly available.

When an article is published we post the peer reviewers' comments and the authors' responses online. We also post the versions of the paper that were used during peer review. These are the versions that the peer review comments apply to.

The versions of the paper that follow are the versions that were submitted during the peer review process. They are not the versions of record or the final published versions. They should not be cited or distributed as the published version of this manuscript.

BMJ Open is an open access journal and the full, final, typeset and author-corrected version of record of the manuscript is available on our site with no access controls, subscription charges or pay-per-view fees (http://bmjopen.bmj.com).

If you have any questions on BMJ Open's open peer review process please email info.bmjopen@bmj.com

BMJ Open

An economic evaluation of a specialist social worker role in reducing length of stay for medically stable inpatients with complex psychosocial needs

Journal:	*BMJ Open*
Manuscript ID	bmjopen-2018-023127
Article Type:	Research
Date Submitted by the Author:	27-Mar-2018
Complete List of Authors:	Osborne, Sonya; Queensland University of Technology, Australian Centre for Health Services Innovation<br>Harrison, Gai; Royal Brisbane and Women's Hospital<br>O'Malia, Angela; Royal Brisbane and Women's Hospital, Department of Social Work and Psychology<br>Barnett, Adrian; Queensland University of Technology, Institute of Health and Biomedical Innovation<br>Carter, Hannah; QUT, AusHSI<br>Graves, Nicholas; QUT, IHBI
Keywords:	Social work, Long stay patient, Economic evaluation, Cost, Discharge barriers

SCHOLARONE™
Manuscripts

TITLE PAGE

Title

An economic evaluation of a specialist social worker role in reducing length of stay for medically stable inpatients with complex psychosocial needs.

Authors

Sonya Osborne | Australian Centre for Health Services Innovation | Queensland University of Technology | Faculty of Health | School of Public Health and Social Work | Kelvin Grove (Brisbane), Queensland 4059 Australia | s.osborne@qut.edu.au

Gai Harrison |Royal Brisbane & Women's Hospital | Department of Social Work |Herston QLD 4029 Australia | gai.harrison@health.qld.gov.au

Angela O'Malia |Royal Brisbane & Women's Hospital | Department of Social Work |Herston QLD 4029 Australia | Angela.OMalia@health.qld.gov.au

Adrian G Barnett | Australian Centre for Health Services Innovation | Queensland University of Technology | Faculty of Health | School of Public Health and Social Work | Kelvin Grove (Brisbane), Queensland 4059 Australia | a.barnett@qut.edu.au

Hannah E Carter* | Australian Centre for Health Services Innovation | Queensland University of Technology | Faculty of Health | School of Public Health and Social Work | Kelvin Grove (Brisbane), Queensland 4059 Australia | hannah.carter@qut.edu.au

Nicholas Graves | Australian Centre for Health Services Innovation | Queensland University of Technology | Faculty of Health | School of Public Health and Social Work | Kelvin Grove (Brisbane), Queensland 4059 Australia | n.graves@qut.edu.au

*Corresponding Author

Word count: 2,879

For peer review only

ABSTRACT

Objective: The primary objective was to determine the impact of a specialist social worker-led model of care in reducing lengths of stay for medically stable patients with complex psychosocial needs who were at risk for long stay. The secondary objective was to determine the economic value of the model of care by estimating the cost per bed day released.

Design: A prospective, matched cohort study with historical controls.

Setting: A large, tertiary teaching and referral hospital in metropolitan Southeast Queensland, Australia.

Methods: Length of hospital stay for a cohort of 53 patients seen under the specialist social worker-led model of care was compared with a matched control group of patients admitted to the hospital prior to the introduction of the new model of care by a survival analysis using a Cox model. An estimate of the "cost per bed day" was produced to inform judgements around the value for money of the model of care.

Results: The model of care reduced mean length of stay by 30%. This translated to 7,165 bed days released over 12 months. The cost to achieve this was estimated to be AUD $229,000 over 12 months. The cost per bed day released was $78, which falls below reported estimates of hospital decision makers' willingness to pay for a bed day to be released for an alternate use.

Conclusions: A specialist social worker-led model of care reduced length of stay at a relatively low cost. This is likely to represent a cost-effective use of hospital resources

For peer review only

STRENGTHS AND LIMITATIONS OF THIS STUDY

- This is the first economic evaluation of a hospital based social work initiative;
- The small sample size may have contributed to relatively high levels of uncertainty in the results;
- The use of historical controls may have introduced bias or confounding effects beyond those that were controlled for;
- Additional benefits of the model of care, including the potential for improved patient outcomes as well as a reduced workload for existing social workers, were not included in our analysis.

For peer review only

INTRODUCTION

Patients classified as 'long stay' in acute hospitals are at risk of poor health outcomes and use up valuable hospital resources [1-3]. A recent retrospective study of over 22,000 patients concluded that patients staying in hospital longer than 14 days suffered increased morbidity and mortality [4]. Deconditioning and loss of mobility are common outcomes for inpatients who are medically stable but remain in hospital, with older people being particularly at risk [5]. Older people's mobility, muscle strength and aerobic capacity can be adversely affected by just ten days of bed rest, which, alarmingly, translates into a loss of ten years of life [6]. In recognition of these poor health outcomes, hospitals strive to ensure that patients are discharged in a timely manner once they are deemed medically stable.

The prolonged hospitalisation of medically stable patients is often the result of non-medical or psychosocial barriers to discharge [7]. Patients at risk of long stay commonly present with complex psychosocial needs. Some long stay patients remain in hospital because they lack family support, have limited access to community services, or are waiting to be placed in an interim or long term care facility [8-10]. Making the unanticipated transition from hospital to institutional care represents a significant life event for many patients and their families, with associated financial and personal costs that may delay discharge. Patients assessed as no longer having capacity to make decisions about their care may similarly face an extended period of hospitalisation, especially if a guardian needs to be appointed to manage their affairs[11]. Material and social disadvantage such as homelessness are also barriers to discharge, while organisational inefficiencies and lack of residential care options may result in placement delays [12 13]. Without timely intervention, patients who present with complex social needs and vulnerabilities are at risk of being stranded in hospital [13].

Typically, long stay patients or patients at risk of long stay are referred to hospital social workers, who are spending increasing amounts of time working with this patient cohort to address discharge barriers[9 14-16]. However, there is limited evidence on the economic value of hospital social work services and no previous economic evaluations of social work initiatives could be identified.

The purpose of this paper is to report on the economic evaluation of an innovative specialist social worker-led model of care to identify and coordinate timely discharge of patients at risk or actually meeting the criteria of a long stay patient in a large teaching hospital. We used the Consolidated Health Economic Evaluation Reporting Standards (CHEERS) as our reporting guide[17].

For peer review only - http://bmjopen.bmj.com/site/about/guidelines.xhtml

METHODS

Study design, setting and participants

A prospective cohort study with historical controls design was conducted in a large 929 bed, tertiary teaching and referral hospital in metropolitan Southeast Queensland, Australia to evaluate the impact of a specialist social worker-led model of care. The primary objective was to determine the impact on lengths of stay for medically stable patients with complex psychosocial needs who were at risk for long stay. The secondary objective was to determine the economic value of the model of care by estimating the cost per bed day released. We took the perspective of the hospital decision maker. The study was approved by the relevant Human Research Ethics Committees for the hospital site (HREC/16/QRBW/476) and the university (QUT1600001102).

The patient cohort included all patients who were entered onto a purpose-built clinical case management database called Pathfinder and managed by the specialist social worker over a three month period from May to August 2016. A historical control group was assembled from hospital administrative databases by selecting a cohort of patients who received usual care before the specialist social worker-led model of care commenced. In order to select a similar group of patients, control patients were those in acute or subacute wards who had received a social work intervention and met a set of pre-identified criteria determining their risk of long stay. Four controls were generated per case providing 86% power to detect a 40% decrease in length of stay assuming a two-sided 5% type 1 error.

Usual Model of Care

Patients identified as complex long stay patients were managed by individual ward social workers, with varying levels of experience, within their existing caseload of approximately 40 cases at any one time. Social workers were supported and guided to find appropriate solutions to complex discharge barriers through monthly clinical supervision sessions. Processes for early identification and escalation of at-risk patients were ad hoc, with little relationships with key stakeholders and rudimentary data collection to measure key performance indicators.

Specialist Social Worker-led (New) Model of Care

In addition to the individual case management by ward social workers, an additional level of specialised service delivery was added to enhance individual social worker support and expedite discharge through a multi-faceted approach. Key components of the specialist service are listed below, with further detail in Supplementary File 1:

For peer review only - http://bmjopen.bmj.com/site/about/guidelines.xhtml

(1) a highly trained and skilled specialist social worker to undertake an overarching advisory role;

(2) a social worker assistant to support the specialist social worker, particularly to manage administrative tasks;

(3) the development of a clinical case management and reporting database called 'Pathfinder' to allow the specialist social worker to design tailored interventions for each patient based on their unique barriers to discharge, and to monitor, track, manage, and oversee all patients on the residential aged care, adults with disability, and medico-legal pathways;

(4) change in communication and reporting processes (e.g. ward social nurses provided regular progress updates to the social worker assistant who recorded the information into Pathfinder; and the specialist social worker regularly communicated through daily reports to hospital executive and other major stakeholders);

(5) development of clear internal escalation processes for 'stranded patients' to engage hospital executives in making timely and appropriate decisions about patient movement and transfer;

(6) implementation of pathways and partnerships with key agencies and care providers to ensure timely and appropriate discharge, including the development of a nursing home vacancy register;

(7) more robust data collection on key performance indicators.

Statistical and Economic Analysis

Characteristics of the specialist social worker-led model of care cohort and the control group were compared using cross-tabulations and summary statistics. Differences between the two groups were tested using the t-test for continuous variables like age, and a chi-squared test for categorical variables like gender. Fisher's exact test was used in place of the chi-squared test when there were small cell sizes. All analyses were made using the R software package (version 3.1.0).

The difference in length of stay between the 53 patients treated under the new model of care and the control group of patients was estimated using survival analysis, using a Cox model [18] with day of admission as the time scale. The model adjusted for differences in acuity, age, gender and diagnosis between the two groups. Because of the strong positive skew in both costs and length of stay data, these variables were both log-transformed. This means the results are on the multiplicative scale and so differences between groups are presented as a percent change.

To avoid "length bias" from including the days before the patient became a long-stay patient, the new model of care was fitted as a time-dependent variable depending on the day of admission that

For peer review only

each patient was identified as being at risk of a long stay.[19] The key results were the hazard ratios and 95% confidence intervals for discharge. If the lower confidence interval for the new model of care is greater than 1 then it was associated with a reduced length of stay as it speeds the time to discharge. To estimate the reduction in length of stay we used the models developed by Beyersmann, Allignol, and Schumacher (2011)[20] based on state space models with the new model of care as an intermediate state between admission and discharge.

The costs associated with the new model of care are reported in 2017 Australian dollars and include one full-time equivalent specialist social worker for 12 months at a total employment cost of $158,000, plus one full time equivalent social work assistant for 12 months at a total employment cost of $71,000. These costs were assumed to be known with certainty. Discounting of costs was not necessary as the reported time horizon was 12 months only. The activity of the team was reported to be in a range of 20 to 30 patients managed per month, and to model this we used a uniform distribution between 20 and 30. One thousand simulations were made from all distributions to show the uncertainty in the results. An estimate of the “cost per bed day” was produced to inform judgements around the value for money of the new model of care. This metric recognises that the value of a bed day released may differ according to the decision maker[21].

Patient and public involvement:

The study did not involve contact with individual patients. Patients were not involved in setting the research question or the outcome measures, nor were they involved in the design and implementation of the study. Given the vulnerability of the patient cohort we do not plan to disseminate results directly to patients.

RESULTS

A total of 204 patients were included; 53 patients in the specialist social worker led model of care group and 151 patients in the control group. As presented in Table 1, there were some differences between the two groups. The new model of care group was older, with age range 32 to 99 years, than the control group, age range 17 to 95 years, with a mean difference of –12 years (95% CI = –17 to –7 years, $p < 0.001$). The new model of care group were almost exclusively from medical wards, whereas 60% of the patients in the control group were from surgical wards (Fisher's exact p-value < 0.001). The ratio of men to women was similar in the two groups (Chi-squared test p-value 0.65).

For peer review only - http://bmjopen.bmj.com/site/about/guidelines.xhtml

Table 1. Participant Demographics

	Usual model of care (n=151)	Specialist social worker-led model of care (n=53)
**Age, mean (S.D.)**	62 (18)	74 (14)
**Gender, n (%)**		
Female	61(40)	24(45)
Male	90(60)	29(55)
**Service/wards**		
Internal Medicine	60(40)	51(96)
Surgery	91(60)	1(2)
Missing	-	1(2)

S.D. = standard deviation

The average length of stay for the entire sample was 65 days and ranged from 3 days to 422 days. The length of stay during the specialist social-led worker period was 23% lower than baseline (95% CI = –42 to 3, p=0.075). After adjusting for gender, age and acute wards, the specialist social worker-led model of care reduced the length of stay by 30% (95% CI –46% to –9%, p=0.007) as presented in Table 2.

Table 2 Multiple regression model of LOS adjusting for gender, age and ward type

**Variable**	Percent Change (Mean)	95% CI		P-value
		Lower	Upper	
Social worker intervention	-30	-46	-9	0.007
Gender (male)	-2	-19	19	0.868
Age (+ 10 years)	-6	-12	-1	0.032
Ward (surgical)	-26	-41	-6	0.013
Ward (missing)	6	-73	321	0.931

CI = confidence interval

The mean number of bed days expected to be released per patient managed under the new model of care was 24. The distribution around this result was skewed and ranged from 0 to 111 over 1,000 Monte Carlo simulations (Figure 1).

The cost to run the model of care for 12 months was $228,876. Based on the modelled simulations, a total of 7,165 bed days were released over 12 months at an average cost of $78 per bed day released (Table 3).

For peer review only

Table 3 Results of 1,000 Monte Carlo simulations from the input parameter distributions

	Mean	S.D.	Min	Max
Total bed days released per patient managed	24	18	0	111
Average number of patients managed per month	25	3	20	30
Total bed days released per year	7,165	5,780	78	38,669
Average cost to save one bed day	$78	$167	$6	$2,932

S.D = standard deviation

Recent data from a survey of Australian hospital CEOs revealed on average they are willing to pay $216 for a ward bed day released from a quality improving activity[21]. Applying this finding, if the decision makers are willing to pay $216 to release a bed day there is a 95% probability (952/1000 simulations) this model of care will meet the decision criterion and only a 5% chance that adopting the model of care was the wrong decision. There was an 82% probability (820/1000 simulations) that the cost of saving one bed day was less than $100.

DISCUSSION

This is the first study to report on the economic evaluation of a hospital based social work initiative. We found a specialist social worker-led model of care reduced the average length of stay by 30%, with 7,165 bed days released over 12 months. This translated to a cost of $78 per bed day released. By definition, this study cannot be considered a cost-effectiveness analysis as we did not explicitly account for the value of patients' health outcomes. However, length of stay is now a well-recognised measure of resource use and efficiency in hospitals, with delayed discharge resulting in reduced patient flow and 'access block' for new admissions[22]. Applying the findings of Page et al [21] that hospital decision makers are willing to pay up to $216 to release one bed day, our results indicate there was a 95% chance that adopting the specialist social worker-led model of care described in this study was the right decision, and by extension likely to represent a cost-effective use of resources. This result is supported by the reality that the study hospital has now formally adopted and funded this model of care on a permanent basis.

Hospitals across Australia and overseas face the challenge of identifying patients at risk of prolonged hospitalisation and addressing barriers to discharge[2 3 13 15]. The foremost imperative for timely discharge is the detrimental affect prolonged hospitalisation has on patient health. However, long stay patients also consume valuable health care resources in an environment where many hospitals face increasing financial constraints. In industrialised nations, up to a third of hospital bed days are attributed to patients who do not require acute care [13]. Bed audits in acute hospitals in the UK have revealed that up to 50% of patients do not require acute care[23]. It has been well documented that

For peer review only

long stay patients commonly present with complex psychosocial needs which can present barriers to discharge [9 11-13].

Although the tendency is for hospital social workers to be referred the most complex patients requiring time-intensive interventions[15], hospital-based social workers are under increased pressure to demonstrate that the services they deliver are both effective and cost-efficient[9 14 15 24]. Notably there has been very limited research conducted on the economic value of social work in hospitals. Auerbach, Mason and Hefte LaPorte described the 'cost-containment' value of social work, which they defined as how effective social workers are 'in both serving patients and keeping hospital costs down'[14]. A 2001 study reported on a modelled cost-benefit analysis of social work services in the emergency department (ED) setting, and found that dedicated social work staffing of EDs may yield net economic benefits to a hospital system, especially in large urban centres[25]. However, a literature review did not identify any economic evaluations of hospital based social work initiatives. This may be a reflection of the inter-personal nature of the profession in which outcomes have traditionally been measured subjectively or qualitatively. However, as hospital decision making becomes increasingly focussed on the provision of high value care[26 27], it is important for the social work discipline to demonstrate the economic, as well as clinical, value of their work.

There are some limitations of the study to note. The cohort seen under the specialist social worker-led model of care was older than the control cohort and contained fewer patients from surgical wards. These differences may have introduced selection bias in the length of stay results. In addition, while the regression model controlled for gender, age and ward type, there may have been other confounders that were not taken into account. The sample size of the model of care cohort was also relatively small which contributed to the uncertainty in cost per bed day results. Not included in this analysis was the potential for the specialist social worker-led model of care to lead to improved patient clinical and quality of life outcomes, as well as a reduced workload for the existing social workers employed by the hospital.

The study was undertaken at a large metropolitan hospital where an oversight role was warranted by the volume of patients being seen. It is unknown whether the efficiencies generated by this role would be replicable in smaller hospital settings. However, the model is scalable with a number of discrete elements that can be adopted separately. There has been interest from other health services in this new model and to date some elements of the model have been adopted by two regional hospitals and one interstate urban hospital.

For peer review only

In conclusion, we found that a specialist social worker-led model of care reduced length of stay in an acute hospital setting at a cost that has been reported as acceptable to hospital decision makers. The model of care is therefore likely to represent a cost-effective use of hospital resources.

For peer review only

ACKNOWLEDGEMENTS

We acknowledge Sarah Napier, who was instrumental in developing the specialist social worker led model of care for long stay patients at RBWH, along with Laura Farrelly and Helen Jones who assisted with data collection.

DECLARATIONS

Ethics approval and consent to participate

The study was approved by the relevant Human Research Ethics Committees for the hospital (HREC/16/QRBW/476) and the university (QUT1600001102).

Consent for publication

All authors have provided consent for publication of this manuscript.

Competing interests

There are no competing interests to declare.

Funding

Funding in the form of in-kind support was provided under a partnership agreement between the study hospital and the Australian Centre for Health Services Innovation (AusHSI).

Authors' contributions

GH and AO'M made substantial contributions to conception and design of the study; AB, HC, SO provided critical review and contributed feedback on the final protocol and ethics application. AB conceived the statistical analysis plan and conducted statistical analysis and interpretation of data; HC conceived the economic analysis plan; NG contributed to the economic data analysis plan and conducted the economic evaluation and interpretation of data. SO and HC drafted the final manuscript and critically revised for important intellectual content. All authors read, revised and approved the final manuscript.

Data sharing statement

No additional data are available

For peer review only

REFERENCES

1. De Coster C, Bruce S, Kozyrskyi A. Use of acute care hospitals by long-stay patients: who, how much, and why? *Canadian Journal of Aging* 2005;24(Suppl 1):97-106.
2. Freitas A, Silva-Costa T, Lopes F, et al. Factors influencing hospital high length of stay outliers. *BMC Health Serv Res* 2012;12:265. doi: 10.1186/1472-6963-12-265
3. Rosman M, Rachminov O, Segal O, et al. Prolonged patients' In-Hospital Waiting Period after discharge eligibility is associated with increased risk of infection, morbidity and mortality: a retrospective cohort analysis. *BMC Health Serv Res* 2015;15:246. doi: 10.1186/s12913-015-0929-6
4. O'Sullivan K, Martensson J, Robbins R, et al. Epidemiology of long-stay patients in a university teaching hospital. *Intern Med J* 2017;47(5):513-21. doi: 10.1111/imj.13379
5. Mecocci P, von Strauss E, Cherubini A, et al. Cognitive impairment is the major risk factor for development of geriatric syndromes during hospitalization: results from the GIFA study. *Dement Geriatr Cogn Disord* 2005;20(4):262-9. doi: 10.1159/000087440
6. Kortebein P, Brock Symons T, Ferrando A, et al. Functional impact of 10 days of bed rest in healthy older adults. *The Journals of Gerontology Series A: Biological Sciences and Medical Sciences* 2008;63(10):1076-81.
7. Jankowski J, Seastrum T, Swidler RN, et al. For lack of a better plan: a framework for ethical, legal, and clinical challenges in complex inpatient discharge planning. *HEC Forum* 2009;21(4):311-26. doi: 10.1007/s10730-009-9117-6
8. Sulman J, Rosenthal CJ, Marshall VW, et al. Elderly Patients in the Acute Care Hospital. *Journal of Gerontological Social Work* 1996;25(3-4):33-52. doi: 10.1300/j083v25n03_04
9. Redfern H, Burton J, Lonne B, et al. Social Work and Complex Care Systems: The Case of People Hospitalised with a Disability. *Australian Social Work* 2015;69(1):27-38. doi: 10.1080/0312407x.2015.1035295
10. Scott IA. Public hospital bed crisis: too few or too misused? . *Australian Health Review* 2010;34(3):317-24.
11. MacKenzie TD, Kukolja T, House R, et al. A discharge panel at Denver Health, focused on complex patients, may have influenced decline in length-of-stay. *Health Aff (Millwood)* 2012;31(8):1786-95. doi: 10.1377/hlthaff.2012.0515
12. Foer D, Ornstein K, Soriano TA, et al. Nonmedical factors associated with prolonged hospital length of stay in an urban homebound population. *J Hosp Med* 2012;7(2):73-8. doi: 10.1002/jhm.992
13. Salonga-Reyes A, Scott IA. Stranded: causes and effects of discharge delays involving non-acute in-patients requiring maintenance care in a tertiary hospital general medicine service. *Aust Health Rev* 2017;41(1):54-62. doi: 10.1071/AH15204
14. Auerbach C, Mason SE, Heft Laporte H. Evidence that supports the value of social work in hospitals. *Soc Work Health Care* 2007;44(4):17-32. doi: 10.1300/J010v44n04_02
15. Galati M, Wong HJ, Morra D, et al. An evidence-based case for the value of social workers in efficient hospital discharge. *Health Care Manag (Frederick)* 2011;30(3):242-6. doi: 10.1097/HCM.0b013e318225e1dd

For peer review only

16. Sims-Gould J, Byrne K, Hicks E, et al. "When Things Are Really Complicated, We Call the Social Worker": Post-Hip-Fracture Care Transitions for Older People. *Health & Social Work* 2015;40(4):257-65. doi: 10.1093/hsw/hlv069
17. Husereau D, Drummond M, Petrou S, et al. Consolidated Health Economic Evaluation Reporting Standards (CHEERS) statement. *BMJ* 2013;346:f1049. doi: 10.1136/bmj.f1049
18. Collett D. Modelling survival data in medical research. 3 ed. London: CRC Press 2014.
19. Wolkewitz M, Allignol A, Harbarth S, et al. Time-dependent study entries and exposures in cohort studies can easily be sources of different and avoidable types of bias. *J Clin Epidemiol* 2012;65(11):1171-80. doi: 10.1016/j.jclinepi.2012.04.008
20. Beyersmann J, Allignol A, Schumacher M. Competing risks and multistate models with R. New York: Springer 2011.
21. Page K, Barnett AG, Graves N. What is a hospital bed day worth? A contingent valuation study of hospital Chief Executive Officers. *BMC health services research* 2017;17(1)
22. Qin S, Thompson C, Bogomolov T, et al. Hospital occupancy and discharge strategies: a simulation-based study. *Intern Med J* 2017;47(8):894-99. doi: 10.1111/imj.13485
23. Monitor. Moving Healthcare closer to home: Literature review of clinical impacts, 2015.
24. Lechman C, Duder S. Psychosocial severity, length of stay and the role of social work services. *Soc Work Health Care* 2006;43(4):1-13. doi: 10.1300/J010v43n04_01
25. Gordon JA. Cost–Benefit Analysis of Social Work Services in the Emergency Department: A Conceptual Model. *Academic Emergency Medicine* 2001;8(1):54-60.
26. CK C, JA G. Choosing wisely: helping physicians and patients make smart decisions about their care. *JAMA* 2012;307(17):1801-2.
27. DK O, A Q, R C, et al. High-value, cost-conscious health care: concepts for clinicians to evaluate the benefits, harms, and costs of medical interventions. *Annals of internal medicine* 2011;154(3):174-80.

For peer review only

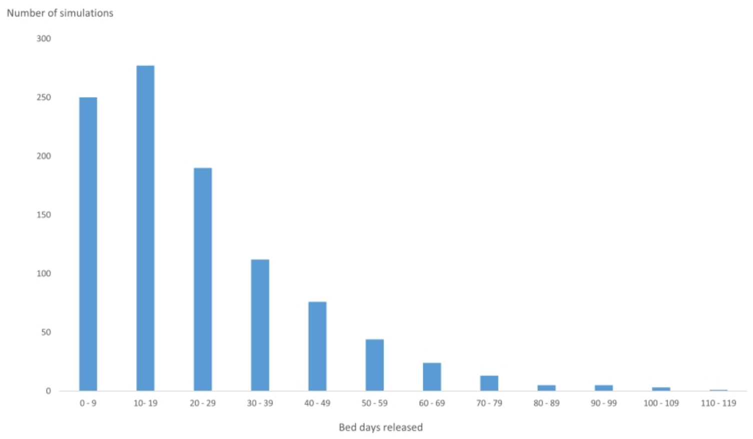

Number of bed days released per patient over 1,000 Monte Carlo simulations

338x190mm (300 x 300 DPI)

Supplementary File: Detailed description of the specialist social worker-led model of care

An innovative specialist social worker-led model of care was developed and piloted in the study site. This social work led model of care took a holistic approach to the management of complex long stay patient discharges and incorporated: micro, patient and family; meso, organisational; and, macro, external strategies and interventions to manage this patient cohort.

Prior to the implementation of this model of care, individual social workers undertook case management responsibility for complex long stay patients as part of their existing caseload within their allocation ward. These social workers had varying levels of experience ranging from newly graduated to experienced; and carried up to 40 cases at any given point. They constantly juggled the demands of immediate discharges with the more specialised requirements of long stay patients. The social workers had little support or guidance beyond monthly clinical supervision sessions to find appropriate solutions to complex discharge barriers, which inherently impacted length of stay. There was ad hoc early identification with no clear escalation processes. There were little to no relationships with key stakeholders and data collection was rudimentary.

In contrast, the new model of care added an additional level of specialised service delivery which was designed to support and enhance the existing social worker service which continued to undertake 'business as usual', but also expedite discharge through a multi-faceted approach. Underpinned by essential elements including the use of a highly skilled specialist worker, a single point of contact and robust data collection and analysis, the model of care also incorporated social work assistant support to maximise efficiency and align tasks to skill level. Internally, the social work department developed processes to ensure the senior social worker was aware of the progress of each long stay case. A social work clinical case management and reporting database called 'Pathfinder' was developed to allow the senior social worker to track, manage, oversee and provide visibility of all patients on the residential aged care, adults with disability and medico-legal pathways. Ward social workers were required to give regular progress updates to the social work assistant who recorded this information in Pathfinder. Once lodged on Pathfinder the senior social assessed all cases, and tailored specific interventions for each case based on the unique psychosocial barriers to discharge. The system also allowed the senior social worker to monitor, track and oversee all cases, as well as providing regular communication to hospital executive through daily reports to hospital executive and other major stakeholders to ensure the organisation had visibility of the progress of these cases.

For peer review only

Another key element to the model of care was the development of clear and timely internal escalation processes to engage hospital executive in decision. Protocols for these interventions were formalised and their benefits were promoted to hospital management and staff to ensure they became embedded into clinicians' everyday practice. The senior social worker, in consultation with the Director of social work escalated stalled cases or 'stranded patients' that posed a significant discharge delay or reputational risk to the organisation due to external institutional delays, family refusal of care, or legal issues.

Externally the model of care implemented pathways and partnerships with key agencies and care providers to ensure timely and appropriate discharges. A nursing home vacancy register was developed alongside a targeted strategy to develop key relationships which resulted in priority notification of vacancies and improved outcomes for patients, especially those with challenging behaviours who previous were difficult to place. Relationships and pathways were also developed with key government agencies, improving their understanding of hospital discharge timeframes and ultimately leading to more responsive turnaround on decisions which had previously taken weeks or months.

An important element of this model was the inclusion of a social work assistant who successfully assisted both the senior social worker and also the ward social workers with sourcing nursing home vacancies using the nursing home vacancy register as well as through the relationships developed with facilities. The role also assisted with time consuming administrative tasks particularly for residential aged care placements such as emails, phone calls, paperwork collation and the maintenance of Pathfinder, nursing home vacancy and other databases. This support was essential to the success of the model by allowing the senior social worker the time to provide the necessary consultations and specialist advice.

Lastly, the model of care also drew on the social workers' specialist skills and knowledge to undertake an advisory role thereby influencing both the organisation and external networks through membership on key committees. The role has been instrumental in the development of new policies and services that ultimately improve outcomes for complex long stay patients. An example of this was the development of strategies for advocating for patients' rights in the face of external institutions' abrogation of their responsibilities to patients such as refusal of ongoing care.

Although developed in a large urban hospital, the model has potential to be modified and scaled to meet local needs and can be implemented without the assistance of a support worker.

For peer review only

CHEERS checklist

Section/item	Item No	Recommendation	Reported on page No/line No
Title and abstract			
Title	1	Identify the study as an economic evaluation or use more specific terms such as "cost-effectiveness analysis", and describe the interventions compared.	Page 1 Line 3
Abstract	2	Provide a structured summary of objectives, perspective, setting, methods (including study design and inputs), results (including base case and uncertainty analyses), and conclusions.	Page 2
Introduction			
Background and objectives	3	Provide an explicit statement of the broader context for the study.<br>Present the study question and its relevance for health policy or practice decisions.	Page 4 paras 1-2<br>Page 4 Para 4
Methods			
Target population and subgroups	4	Describe characteristics of the base case population and subgroups analysed, including why they were chosen.	Page 5 Line 11
Setting and location	5	State relevant aspects of the system(s) in which the decision(s) need(s) to be made.	Page 5 Line 3
Study perspective	6	Describe the perspective of the study and relate this to the costs being evaluated.	Page 5 Line 8
Comparators	7	Describe the interventions or strategies being compared and state why they were chosen.	Page 5 Para 3-4
Time horizon	8	State the time horizon(s) over which costs and consequences are being evaluated and say why appropriate.	Page 5 Line 13
Discount rate	9	Report the choice of discount rate(s) used for costs and outcomes and say why appropriate.	Page 7 Line 10
Choice of health outcomes	10	Describe what outcomes were used as the measure(s) of benefit in the evaluation and their relevance for the type of analysis performed.	Page 6 Line 23
Measurement of effectiveness	11b	*Synthesis-based estimates:* Describe fully the methods used for identification of included studies and synthesis of clinical effectiveness data.	NA
Measurement and valuation of preference based outcomes	12	If applicable, describe the population and methods used to elicit preferences for outcomes.	NA

For peer review only

Section/item	Item No	Recommendation	Reported on page No/line No
Estimating costs and resources	13b	*Model-based economic evaluation:* Describe approaches and data sources used to estimate resource use associated with model health states. Describe primary or secondary research methods for valuing each resource item in terms of its unit cost. Describe any adjustments made to approximate to opportunity costs.	NA
Currency, price date and conversion	14	Report the dates of the estimated resource quantities and unit costs. Describe methods for adjusting estimated unit costs to the year of reported costs if necessary. Describe methods for converting costs into a common currency base and the exchange rate.	Page 7 Line 7
Choice of model	15	Describe and give reasons for the specific type of decision-analytical model used. Providing a figure to show model structure is strongly recommended.	NA
Assumptions	16	Describe all structural or other assumptions underpinning the decision-analytical model.	NA
Analytical methods	17	Describe all analytical methods supporting the evaluation. This could include methods for dealing with skewed, missing, or censored data; extrapolation methods; methods for pooling data; approaches to validate or make adjustments (such as half cycle corrections) to a model; and methods for handling population heterogeneity and uncertainty.	Page 6, line 18 to Page 7, line 16
Results			
Study parameters	18	Report the values, ranges, references, and, if used, probability distributions for all parameters. Report reasons or sources for distributions used to represent uncertainty where appropriate. Providing a table to show the input values is strongly recommended.	Page 7 Line 7
Incremental costs and outcomes	19	For each intervention, report mean values for the main categories of estimated costs and outcomes of interest, as well as mean differences between the comparator groups. If applicable, report incremental cost-effectiveness ratios.	Page 8
Characterising uncertainty	20b	*Model-based economic evaluation:* Describe the effects on the results of uncertainty for all input parameters, and uncertainty related to the structure of the model and assumptions.	NA
Characterising heterogeneity	21	If applicable, report differences in costs, outcomes, or cost-effectiveness that can be explained by variations between subgroups of patients with different baseline characteristics or other observed variability in effects that are not reducible by more information.	Page 8 Line 1 Table 2
Discussion			

For peer review only

Section/item	Item No	Recommendation	Reported on page No/line No
Study findings, limitations, generalisability, and current knowledge	22	Summarise key study findings and describe how they support the conclusions reached. Discuss limitations and the generalisability of the findings and how the findings fit with current knowledge.	Page 9 Line 8 Page 10 Para 3-4
Other			
Source of funding	23	Describe how the study was funded and the role of the funder in the identification, design, conduct, and reporting of the analysis. Describe other non-monetary sources of support.	Page 12 Line 13
Conflicts of interest	24	Describe any potential for conflict of interest of study contributors in accordance with journal policy. In the absence of a journal policy, we recommend authors comply with International Committee of Medical Journal Editors recommendations.	Page 12 Line 11

For peer review only

BMJ Open

Examining the impact of a specialist social worker intervention on length of stay for long stay patients: a cost consequence analysis

Journal:	*BMJ Open*
Manuscript ID	bmjopen-2018-023127.R1
Article Type:	Research
Date Submitted by the Author:	13-Aug-2018
Complete List of Authors:	Osborne, Sonya; Queensland University of Technology, Australian Centre for Health Services Innovation<br>Harrison, Gai; Royal Brisbane and Women's Hospital<br>O'Malia, Angela; Royal Brisbane and Women's Hospital, Department of Social Work and Psychology<br>Barnett, Adrian; Queensland University of Technology, Institute of Health and Biomedical Innovation<br>Carter, Hannah; QUT, AusHSI<br>Graves, Nicholas; QUT, IHBI
**Primary Subject Heading**:	Health services research
Secondary Subject Heading:	Health economics
Keywords:	Social work, Long stay patient, Economic evaluation, Cost, Discharge barriers, Length of stay

SCHOLARONE™ Manuscripts

TITLE PAGE

Title

Examining the impact of a specialist social worker intervention on length of stay for long stay patients: a cost consequence analysis

Authors

Sonya Osborne | Australian Centre for Health Services Innovation | Queensland University of Technology | Faculty of Health | School of Public Health and Social Work | Kelvin Grove (Brisbane), Queensland 4059 Australia | s.osborne@qut.edu.au

Gai Harrison |Royal Brisbane & Women's Hospital | Department of Social Work |Herston QLD 4029 Australia | gai.harrison@health.qld.gov.au

Angela O'Malia |Royal Brisbane & Women's Hospital | Department of Social Work |Herston QLD 4029 Australia | Angela.OMalia@health.qld.gov.au

Adrian G Barnett | Australian Centre for Health Services Innovation | Queensland University of Technology | Faculty of Health | School of Public Health and Social Work | Kelvin Grove (Brisbane), Queensland 4059 Australia | a.barnett@qut.edu.au

Hannah E Carter* | Australian Centre for Health Services Innovation | Queensland University of Technology | Faculty of Health | School of Public Health and Social Work | Kelvin Grove (Brisbane), Queensland 4059 Australia | hannah.carter@qut.edu.au

Nicholas Graves | Australian Centre for Health Services Innovation | Queensland University of Technology | Faculty of Health | School of Public Health and Social Work | Kelvin Grove (Brisbane), Queensland 4059 Australia | n.graves@qut.edu.au

*Corresponding Author

Word count: 2,879

For peer review only - http://bmjopen.bmj.com/site/about/guidelines.xhtml

ABSTRACT

Background: Long stay patients in acute hospitals commonly present with complex psychosocial needs. Hospital based social workers are spending increasing amounts of time working with this patient cohort to address barriers to discharge.

Objective: The primary objective was to determine the impact of a specialist social worker-led model of care in reducing lengths of stay for medically stable patients with complex psychosocial needs who were at risk for long stay. The secondary objective was to determine the economic value of the model of care by estimating the cost per bed day avoided relative to the decision makers' willingness to pay for bed days released.

Design: A prospective, matched cohort study with historical controls.

Setting: A large, tertiary teaching and referral hospital in metropolitan Southeast Queensland, Australia.

Methods: Length of hospital stay for a cohort of patients seen under the specialist social worker-led model of care was compared with a matched control group of patients admitted to the hospital prior to the introduction of the new model of care using a multistate model with the social worker model of care as an intermediate event. Costs associated with the model of care were calculated and an estimate of the "cost per bed day" was produced.

Results: The model of care reduced mean length of stay by 33 days. This translated to 9,999 bed days released over 12 months. The cost to achieve this was estimated to be AUD $229,000 over 12 months. The cost per bed day released was $23, which is below estimates of hospital decision makers' willingness to pay for a bed day to be released for an alternate use.

Conclusions: A specialist social worker-led model of care reduced length of stay at a relatively low cost. This is likely to represent a cost-effective use of hospital resources.

For peer review only - http://bmjopen.bmj.com/site/about/guidelines.xhtml

STRENGTHS AND LIMITATIONS OF THIS STUDY

- This is the first economic evaluation of a hospital based social work initiative;
- The small sample size contributed to relatively high levels of uncertainty in the results;
- The use of historical controls may have introduced bias or confounding effects beyond those that were controlled for;
- Additional benefits of the model of care, including the potential for improved patient outcomes as well as a reduced workload for existing social workers, were not included in our analysis.

For peer review only

INTRODUCTION

The longer patients remain in hospital after they are medically ready for discharge, the worse the situation becomes for the patient, their families, and for the health system. Patients classified as 'long stay' in acute hospitals are at risk of poor health outcomes and use up valuable hospital resources [1-3]. A retrospective observational study of over 22,000 patients concluded that patients staying in hospital 14 days or longer suffered increased in-hospital morbidity and mortality [4]. Deconditioning and loss of mobility are common outcomes for inpatients who are medically fit for discharge but remain in hospital, with older people being particularly at risk [5]. For patients aged 65 or older, length of stay increased the chance of developing geriatric syndromes in hospital, such as pressure ulcers, incontinence, falls, functional decline and delirium; independent of physical, cognitive or functional impairment [5]. In addition, older people's mobility, muscle strength and aerobic capacity can be adversely affected by just ten days of bed rest, which, alarmingly, translates into a loss of ten years of life [6]. In recognition of these poor health outcomes, hospitals strive to ensure that patients are discharged in a timely manner once they are deemed medically stable, or fit for discharge, that is, once the medical decision has been made that the patient has completed the required acute care, including all relevant investigations with none further anticipated, and is ready to be discharged from the hospital.

Long stay, that is, the prolonged hospitalisation of patients who are medically fit for discharge, is often the result of non-medical or complex psychosocial barriers to discharge. Patients at risk of long stay commonly present with complex psychosocial needs [7]. Some long stay patients remain in hospital because they lack family support, have limited access to community services, or are waiting to be placed in an interim or long-term care facility [8-10]. Making the unanticipated transition from hospital to institutional care represents a significant life event for many patients and their families, with associated financial and personal costs that may delay discharge. Patients assessed as no longer having capacity to make decisions about their care may similarly face an extended period of hospitalisation, especially if a guardian needs to be appointed to manage their affairs[11]. Material and social disadvantage such as homelessness are also barriers to discharge, while organisational inefficiencies and lack of residential care options may result in placement delays [12 13]. Without timely intervention, patients who present with complex social needs and vulnerabilities are at risk of being stranded in hospital [13].

Typically, long stay patients are referred to hospital social workers, who are spending increasing amounts of time working with this patient cohort to address discharge barriers[9 14-16]. However, there

For peer review only - http://bmjopen.bmj.com/site/about/guidelines.xhtml

is limited evidence on the economic value of hospital social work services, and even less evidence on the economic value of social work initiatives targeting long stay patients.

The purpose of this paper is to report on an economic evaluation of an innovative specialist social worker-led model of care designed to identify and coordinate timely discharge of long stay patients and those at risk of a long stay in a large teaching hospital. We estimated the cost of delivering the model of care and reported its benefits in terms of bed days released. An assessment of the economic value of the intervention was made using previously reported estimates of Australian hospital decision makers' maximum willingness to pay for a bed day to be released for an alternate use. We used the Consolidated Health Economic Evaluation Reporting Standards (CHEERS) to guide our study report[17].

METHODS

Establishing a Profile of Long Stay Patients

Prior to developing any intervention to address the issue of long stay patients, it was critical to establish a profile of this vulnerable patient cohort. In March 2016, a snapshot one-day audit of several hospital administrative databases was conducted to identify acute patients whose length of stay was equal to or greater than 21 days and sub-acute patients whose length of stay was equal to or greater than 35 days. Patients in intensive care units, maternity wards, mental health wards, and the emergency department were excluded from the audit. A total of 93 patients met the inclusion criteria. Using a purpose-designed audit tool, two persons, a nurse navigator and a senior social worker, independently reviewed each chart to identify barriers to discharge. Eleven psychosocial barriers were identified as risk factors for long stay. These risk factors included family complexity, dysfunction or conflict; domestic and family violence; need for substitute decision making; carer stress; substance abuse; homelessness; challenging patient behaviours; disability; patient or family financial stress; need for residential aged care placement; and disability care planning. These risk factors were in turn able to inform the early identification of patients at risk of long stay. Further explanations of the psychosocial barriers to discharge and several theoretical case study exemplars of the psychosocial complexities of long stay patients can be found Supplementary Files 1 and 2.

Study design, setting and participants

A prospective cohort study with historical controls design was conducted in a large 929 bed, tertiary teaching and referral hospital in metropolitan Southeast Queensland, Australia to evaluate the impact of a specialist social worker-led model of care. The primary objective was to determine the

For peer review only - http://bmjopen.bmj.com/site/about/guidelines.xhtml

impact of the specialist social worker-led model of care on lengths of stay for patients who were medically stable and fit for discharge before and after introduction of the new model of care. The secondary objective was to determine the economic value of the model of care by estimating the cost per bed day released. We took the perspective of the hospital decision maker and their willingness to pay for a bed day released for an alternate use. The study was approved by the relevant Human Research Ethics Committees for the hospital site (HREC/16/QRBW/476) and the university (QUT1600001102).

The patient cohort included all patients who were entered onto a purpose-built clinical case management database called Pathfinder and managed by the specialist social worker over a three month period from June to August 2016. A historical control group was assembled from hospital administrative databases by selecting a cohort of patients who received usual care before the specialist social worker-led model of care commenced. In order to select a similar group of patients, control patients were those in acute or subacute wards who met the definition of a long stay patient and who had received a social work intervention.

Usual Model of Care (Control)

Prior to the introduction of the new model of care, long stay patients were case managed solely by the allocated ward based social worker who typically held up to 40 cases at any one time of both acute short term and long stay patients. The social work intervention for all cases included psychosocial assessment with view to managing appropriate and timely discharge. Typically referrals for long stay patients were made to social work once the patient was medically stable. There were no early screening processes to identify long stay patients so early intervention was not undertaken. Acute short term patients were often prioritised to maintain rapid patient flow yet there was no framework in place to manage the multiple tasks, meetings, stakeholder liaison, reports and paperwork associated with the care of long stay patients. The service was reactive rather than proactive there was no visibility over delays to social work intervention. Importantly, there was no formal escalation process in place to resolve systemic issues so individual social workers typically attempted to resolve these matters independently, often without success, resulting in lengthy delays.

Specialist Social Worker-led (New) Model of Care (Intervention)

In addition to the individual case management by ward social workers, an additional level of specialised service delivery was added to enhance individual social worker support and expedite discharge through a multi-faceted approach. A process was introduced whereby ward social workers

For peer review only - http://bmjopen.bmj.com/site/about/guidelines.xhtml

could refer patients they believed to be at risk of long stay to the specialist social worker via clinical consultation. These referrals were subsequently entered on an electronic patient tracking system called Pathfinder by the specialist social worker. This allowed the senior social worker to monitor patients at risk of prolonged hospitalisation. The tracking program was a core component of the model of care and was updated daily by ward social workers. As a clinical case management tool, it recorded psychosocial and systemic barriers to discharge as well as current length of stay, which allowed continuous monitoring of patient outcomes. The specialist social worker also regularly collated a list of long stay patients from hospital databases and then consulted with the different social work teams to identify those patients facing psychosocial barriers to discharge. In contrast to the old model of care, the new model was proactive rather than reactive and adopted a systematic approach to identifying and monitoring long stay patients or those at risk of long stay. Key components of the specialist service are listed below, with further detail in Supplementary File 3:

(1) a highly trained and skilled specialist social worker to undertake an overarching advisory role;

(2) a social worker assistant to support the specialist social worker, particularly to manage administrative tasks;

(3) the development of a clinical case management and reporting database called 'Pathfinder' to allow the specialist social worker to design tailored interventions for each patient based on their unique barriers to discharge, and to monitor, track, manage, and oversee all patients on the residential aged care, adults with disability, and medico-legal pathways;

(4) change in communication and reporting processes (e.g. ward social nurses provided regular progress updates to the social worker assistant who recorded the information into Pathfinder; and the specialist social worker regularly communicated through daily reports to hospital executive and other major stakeholders);

(5) development of clear internal escalation processes for 'stranded patients' to engage hospital executives in making timely and appropriate decisions about patient movement and transfer;

(6) implementation of pathways and partnerships with key agencies and care providers to ensure timely and appropriate discharge, including the development of a nursing home vacancy register;

(7) more robust data collection on key performance indicators.

Statistical and Economic Analysis

Characteristics of the specialist social worker-led model of care cohort and the control group were compared using cross-tabulations and summary statistics. Differences between the two groups were

For peer review only - http://bmjopen.bmj.com/site/about/guidelines.xhtml

tested using the t-test for continuous variables like age, and a chi-squared test for categorical variables like gender. All analyses were made using the R software package (version 3.1.0). The difference in length of stay between the patients treated under the new model of care and the control group of patients was estimated using a multistate model.

To estimate the reduction in length of stay we used the models developed by Beyersmann, Allignol, and Schumacher (2011)[18] with the new model of care as an intermediate state between admission and discharge (see Figure 1). Confidence intervals for the length of stay were generated by bootstrapping the data and recalculating the difference in length of stay associated with the social worker intervention. We used 1000 bootstrap estimates.

The bootstrap length of stay estimates were applied in a Monte Carlo simulation to model the cost and length of stay outcomes over a 12 month period. The costs associated with the new model of care are reported in 2017 Australian dollars and include one full-time equivalent specialist social worker for 12 months at a total employment cost of $158,000, plus one full time equivalent social work assistant for 12 months at a total employment cost of $71,000. These costs were assumed to be known with certainty. Discounting of costs was not necessary as the reported time horizon was only 12 months. The activity of the team was reported to be in a range of 20 to 30 patients managed per month, and to model this we used a uniform distribution between 20 and 30. One thousand simulations were made from all distributions to account for the uncertainty in the results. An estimate of the “cost per bed day” was produced to inform judgements around the value for money of the new model of care. This metric recognises that the value of a bed day released may differ according to the decision maker[19]. We used estimates from a recent survey of Australian hospital CEOs which revealed they are willing to pay an average of $216 for a ward bed day released from a quality improving activity[19].

Patient and public involvement:

The study did not involve contact with individual patients. Patients were not involved in setting the research question or the outcome measures, nor were they involved in the design and implementation of the study. Given the vulnerability of the patient cohort we do not plan to disseminate results directly to patients.

For peer review only - http://bmjopen.bmj.com/site/about/guidelines.xhtml

RESULTS

A total of 112 patients were included; 52 patients in the specialist social worker led model of care group and 60 patients in the control group. As presented in Table 1, there were some differences between the two groups. The new model of care group was older, with mean difference of 5 years (95% CI -1 to 11 years, p-value 0.11). The ratio of men to women was similar in the two groups (Chi-squared test p-value 0.66).

Table 1. Participant Demographics

	Usual model of care (n=60)	Specialist social worker-led model of care (n=52)
**Age, mean (S.D.)**	69 (18)	74 (14)
**Gender, n (%)**		
Female	23(38)	23(44)
Male	37(62)	29(56)

S.D. = standard deviation

The average length of stay for the entire sample was 70 days and ranged from 3 days to 422 days. The median time between admission and involvement of the social worker was 25 days, with an inter-quartile range from 9 to 50 days.

The mean number of bed days expected to be released per patient managed under the new model of care was 33. The distribution around this result was skewed and ranged from 5 to 70 over 1,000 Monte Carlo simulations (Figure 2).

The cost to run the model of care for 12 months was $228,876. Based on the modelled simulations, a total of 9,999 bed days were released over 12 months at an average cost of $23 per bed day released (Table 2).

Table 2 Results of 1,000 Monte Carlo simulations from the input parameter distributions

	Mean	S.D.	Min	Max
Total bed days released per patient managed	33	12	5	70
Average number of patients managed per month	25	3	20	30
Total bed days released per year	9,999	3,781	1140	24,408
Average cost to save one bed day	$23	$16	$9	$201

S.D = standard deviation

When applying the finding that decision makers are willing to pay $216 to release a bed day there was a 100% probability (1000/1000 simulations) this model of care will meet the decision criterion.

For peer review only - http://bmjopen.bmj.com/site/about/guidelines.xhtml

There was an 82% probability (820/1000 simulations) that the cost of saving one bed day was less than $100.

DISCUSSION

This is the first study to report on the economic evaluation of a hospital based social work initiative. We found a specialist social worker-led model of care reduced the average length of stay by 33 days, with 9,999 bed days released over 12 months. This translated to a cost of $23 per bed day released. By definition, this study cannot be considered a cost-effectiveness analysis as we did not explicitly account for the value of patients' health outcomes. However, length of stay is now a well-recognised measure of resource use and efficiency in hospitals, with delayed discharge resulting in reduced patient flow and 'access block' for new admissions[20]. Applying the findings of Page et al [19] that hospital decision makers are willing to pay up to $216 to release one bed day, our results indicate there was a 100% chance that adopting the specialist social worker-led model of care described in this study was the right decision, and by extension likely to represent a cost-effective use of resources. This result is supported by the reality that the study hospital has now formally adopted and funded this model of care on a permanent basis.

The new model of care prioritised early identification of patients at risk of long stay, patient tracking, proactive intervention, and system level responses. These four factors promoted a more coordinated and targeted social work response to addressing psychosocial barriers to discharge which in turn assisted in expediting hospital discharge. In addition, early identification of potential long stay patients was facilitated by clinicians' greater awareness of the main psychosocial barriers to discharge when conducting initial psychosocial assessments.

Although the tendency is for hospital social workers to be referred the most complex patients requiring time-intensive interventions[15], hospital-based social workers are under increased pressure to demonstrate that the services they deliver are both effective and cost-efficient[9 14 15 21]. Notably there has been very limited research conducted on the economic value of social work in hospitals. Auerbach, Mason and Hefte LaPorte described the 'cost-containment' value of social work, which they defined as how effective social workers are 'in both serving patients and keeping hospital costs down'[14]. A 2001 study reported on a modelled cost-benefit analysis of social work services in the emergency department (ED) setting, and found that dedicated social work staffing of EDs may yield net economic benefits to a hospital system, especially in large urban centres[22]. However, a literature review did not identify any economic evaluations of hospital based social work initiatives. This may be a reflection of the inter-personal nature of the profession in which outcomes have traditionally

For peer review only - http://bmjopen.bmj.com/site/about/guidelines.xhtml

been measured subjectively or qualitatively. However, as hospital decision making becomes increasingly focussed on the provision of high value care[23 24], it is important for the social work discipline to demonstrate the economic, as well as clinical, value of their work.

The comparability of intervention and control groups is a key weakness of our study. Control patients were selected after having met the definition of long stay, while intervention patients were able to be selected without first meeting this definition. This approach enabled us to best reflect the change in practice that occurred given the retrospective nature of our analysis. Specifically, there was no process for the early identification of patients at risk of long stay prior to the intervention, with ward social workers typically becoming involved once patients had met the definition of long stay and were experiencing barriers to discharge. Following the introduction of the new model of care, a focus on early identification meant that patients could be identified, tracked and monitored prior to them meeting the long stay definition. We acknowledge that these differences may have introduced selection bias in the length of stay results. A prospective pre-post study design with clear criteria about eligible patients and a staff member employed to monitor patients and include them would have allowed for the selection of more comparable controls. The sample size of both cohorts was also relatively small which contributed to the uncertainty in cost per bed day results. Not included in this analysis was the potential for the specialist social worker-led model of care to lead to improved patient clinical and quality of life outcomes, as well as a reduced workload for the existing social workers employed by the hospital.

The study was undertaken at a large metropolitan hospital where an oversight role was warranted by the volume of patients being seen. It is unknown whether the efficiencies generated by this role would be replicable in smaller hospital settings. However, the model is scalable with a number of discrete elements that can be adopted separately. There has been interest from other health services in this new model and to date some elements of the model have been adopted by two regional hospitals and one interstate urban hospital.

In conclusion, we found that a specialist social worker-led model of care reduced length of stay in an acute hospital setting at a cost that has been reported as acceptable to hospital decision makers. The model of care is therefore likely to represent a cost-effective use of hospital resources.

For peer review only - http://bmjopen.bmj.com/site/about/guidelines.xhtml

FIGURE LEGENDS

Figure 1: The three states used to measure the change in length of stay due to the social worker intervention

Figure 2: Histogram of the bed days released per patient due to the social worker intervention

ACKNOWLEDGEMENTS

We acknowledge Sarah Napier, who was instrumental in developing the specialist social worker-led model of care for long stay patients at study hospital, along with Laura Farrelly and Helen Jones who assisted with data collection.

DECLARATIONS

Ethics approval and consent to participate

The study was approved by the relevant Human Research Ethics Committees for the hospital (HREC/16/QRBW/476) and the university (QUT1600001102).

Consent for publication

All authors have provided consent for publication of this manuscript.

Competing interests

There are no competing interests to declare.

Funding

Funding in the form of in-kind support was provided under a partnership agreement between the study hospital and the Australian Centre for Health Services Innovation (AusHSI).

Authors' contributions

GH and AO'M made substantial contributions to conception and design of the study; AB, HC, SO provided critical review and contributed feedback on the final protocol and ethics application. AB conceived the statistical analysis plan and conducted statistical analysis and interpretation of data; HC conceived the economic analysis plan; NG contributed to the economic data analysis plan and conducted the economic evaluation and interpretation of data. SO and HC drafted the final manuscript and critically revised for important intellectual content. All authors read, revised and approved the final manuscript.

For peer review only - http://bmjopen.bmj.com/site/about/guidelines.xhtml

For peer review only

Data Sharing Statement

The full dataset used, which has no identifying information, is available from the corresponding author

For peer review only

For peer review only - http://bmjopen.bmj.com/site/about/guidelines.xhtml

REFERENCES

1. De Coster C, Bruce S, Kozyrskyi A. Use of acute care hospitals by long-stay patients: who, how much, and why? *Canadian Journal of Aging* 2005;24(Suppl 1):97-106.
2. Freitas A, Silva-Costa T, Lopes F, et al. Factors influencing hospital high length of stay outliers. *BMC Health Serv Res* 2012;12:265. doi: 10.1186/1472-6963-12-265
3. Rosman M, Rachminov O, Segal O, et al. Prolonged patients' In-Hospital Waiting Period after discharge eligibility is associated with increased risk of infection, morbidity and mortality: a retrospective cohort analysis. *BMC Health Serv Res* 2015;15:246. doi: 10.1186/s12913-015-0929-6
4. O'Sullivan K, Martensson J, Robbins R, et al. Epidemiology of long-stay patients in a university teaching hospital. *Intern Med J* 2017;47(5):513-21. doi: 10.1111/imj.13379
5. Mecocci P, von Strauss E, Cherubini A, et al. Cognitive impairment is the major risk factor for development of geriatric syndromes during hospitalization: results from the GIFA study. *Dement Geriatr Cogn Disord* 2005;20(4):262-9. doi: 10.1159/000087440
6. Kortebein P, Brock Symons T, Ferrando A, et al. Functional impact of 10 days of bed rest in healthy older adults. *The Journals of Gerontology Series A: Biological Sciences and Medical Sciences* 2008;63(10):1076-81.
7. Jankowski J, Seastrum T, Swidler RN, et al. For lack of a better plan: a framework for ethical, legal, and clinical challenges in complex inpatient discharge planning. *HEC Forum* 2009;21(4):311-26. doi: 10.1007/s10730-009-9117-6
8. Sulman J, Rosenthal CJ, Marshall VW, et al. Elderly Patients in the Acute Care Hospital. *Journal of Gerontological Social Work* 1996;25(3-4):33-52. doi: 10.1300/j083v25n03_04
9. Redfern H, Burton J, Lonne B, et al. Social Work and Complex Care Systems: The Case of People Hospitalised with a Disability. *Australian Social Work* 2015;69(1):27-38. doi: 10.1080/0312407x.2015.1035295
10. Scott IA. Public hospital bed crisis: too few or too misused? . *Australian Health Review* 2010;34(3):317-24.
11. MacKenzie TD, Kukolja T, House R, et al. A discharge panel at Denver Health, focused on complex patients, may have influenced decline in length-of-stay. *Health Aff (Millwood)* 2012;31(8):1786-95. doi: 10.1377/hlthaff.2012.0515
12. Foer D, Ornstein K, Soriano TA, et al. Nonmedical factors associated with prolonged hospital length of stay in an urban homebound population. *J Hosp Med* 2012;7(2):73-8. doi: 10.1002/jhm.992
13. Salonga-Reyes A, Scott IA. Stranded: causes and effects of discharge delays involving non-acute in-patients requiring maintenance care in a tertiary hospital general medicine service. *Aust Health Rev* 2017;41(1):54-62. doi: 10.1071/AH15204
14. Auerbach C, Mason SE, Heft Laporte H. Evidence that supports the value of social work in hospitals. *Soc Work Health Care* 2007;44(4):17-32. doi: 10.1300/J010v44n04_02
15. Galati M, Wong HJ, Morra D, et al. An evidence-based case for the value of social workers in efficient hospital discharge. *Health Care Manag (Frederick)* 2011;30(3):242-6. doi: 10.1097/HCM.0b013e318225e1dd
16. Sims-Gould J, Byrne K, Hicks E, et al. "When Things Are Really Complicated, We Call the Social Worker": Post-Hip-Fracture Care Transitions for Older People. *Health & Social Work* 2015;40(4):257-65. doi: 10.1093/hsw/hlv069
17. Husereau D, Drummond M, Petrou S, et al. Consolidated Health Economic Evaluation Reporting Standards (CHEERS) statement. *BMJ* 2013;346:f1049. doi: 10.1136/bmj.f1049
18. Beyersmann J, Allignol A, Schumacher M. Competing risks and multistate models with R. New York: Springer 2011.

For peer review only - http://bmjopen.bmj.com/site/about/guidelines.xhtml

19. Page K, Barnett AG, Graves N. What is a hospital bed day worth? A contingent valuation study of hospital Chief Executive Officers. *BMC health services research* 2017;17(1)
20. Qin S, Thompson C, Bogomolov T, et al. Hospital occupancy and discharge strategies: a simulation-based study. *Intern Med J* 2017;47(8):894-99. doi: 10.1111/imj.13485
21. Lechman C, Duder S. Psychosocial severity, length of stay and the role of social work services. *Soc Work Health Care* 2006;43(4):1-13. doi: 10.1300/J010v43n04_01
22. Gordon JA. Cost–Benefit Analysis of Social Work Services in the Emergency Department: A Conceptual Model. *Academic Emergency Medicine* 2001;8(1):54-60.
23. Cassel CK, Guest JA. Choosing wisely: helping physicians and patients make smart decisions about their care. *JAMA* 2012;307(17):1801-2. doi: doi:10.1001/jama.2012.476
24. Owens DK, Qaseem A, Chou R, et al. High-value, cost-conscious health care: concepts for clinicians to evaluate the benefits, harms, and costs of medical interventions. *Annals of internal medicine* 2011;154(3):174-80. doi: 10.7326/0003-4819-154-3-201102010-00007

For peer review only

For peer review only - http://bmjopen.bmj.com/site/about/guidelines.xhtml

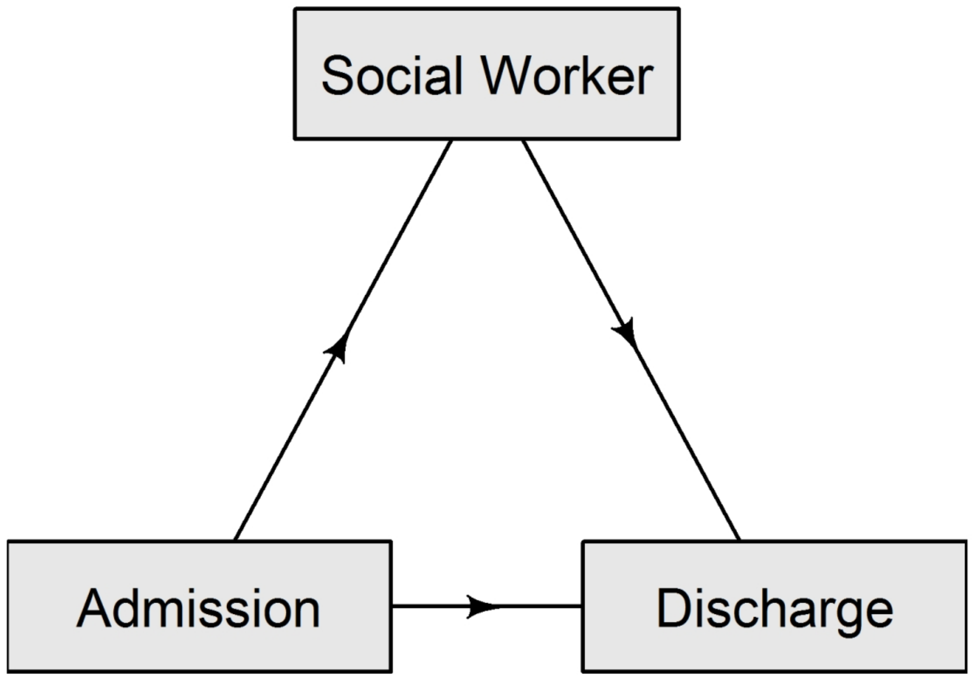

The three states used to measure the change in length of stay due to the social worker intervention

127x101mm (300 x 300 DPI)

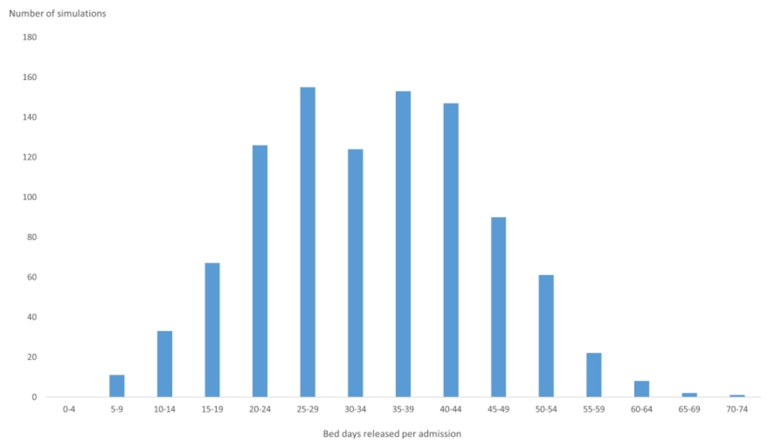

Histogram of the bed days released per patient due to the social worker intervention

338x190mm (300 x 300 DPI)

Supplementary File 1.

Psychosocial Barriers to Discharge Identified from Audit of Long Stay Patients

Barriers to discharge	Examples and/or impact on patient, carer, family
Family complexity (dysfunction or conflict)	Family members may disagree on whether patient should go into residential aged care or go home or disagreeing on care arrangements for a patient post-discharge. This could also include family estrangement, inability to locate family, or situation where the partner of a patient does not want the patient discharged because the partner is currently involved in another relationship.
Domestic and family violence/Elder abuse	This could be suspected or confirmed. It is often difficult to confirm elder abuse, especially if the patient has cognitive impairment, dementia, mental health issues, etc. However, even if abuse cannot be confirmed, the health care professional cannot discount the risk to the patient if he/she is discharged into the care of a suspected abuser.
Substitute decision making	The patient may be awaiting assessment by a civil or administrative tribunal, which is an independent, accessible tribunal that efficiently resolves disputes between parties on a range of matters, such as establishing who is the substitute decision maker if there are complex family dynamics (for example, the patient recently formed a new partnership but has adult children), or the patient has become incapacitated or unable to make sound decisions on their behalf.
Carer stress	Carers indicate that they can no longer manage the demands of caring for the patient and/or are experiencing burnout.
Substance abuse	Patient substance abuse may reduce accommodation options. Substance abuse may also manifest in patient aggression.
Homelessness	For homeless patients, there are usually no viable discharge options.
Challenging behaviours	This is usually in reference to challenging behaviours displayed by the patient, for example, physically or verbally abusive behaviour towards others; not complying with medical care; sexually inappropriate behaviour; dementia related behaviours (e.g. wandering); verbal aggression. These types of behaviours make it harder to find facilities who will accept patients who are likely to impact on the quality of life of other residents or upset staff.
Disability	This usually refers to patients with disabilities where finding suitable out of hospital placements or in-home carers may be difficult.
Financial stress	This could be in relation to the patient and/or the family and could manifest as significant debt or lack of regular income; whereby, patient may not be able to afford residential aged care.
Residential Aged Care	A situation where the patient requires residential care but there is no suitable vacancy or alternatively, the patient may simply be awaiting placement.
Disability Care Planning	A patient may need services at home and be awaiting services at home to be put in place; or there is a lack of suitable accommodation options to meet the disability needs of the patient.

For peer review only

Supplementary File 2. Conceptual Case Study Exemplars[1] of Long Stay Patients with Complex Psychosocial Barriers to Discharge

Case 1 Jack

Jack is an Aboriginal man in his mid-sixties who **at the time of admission had been homeless for several years.** He had previously resided in supported accommodation, but had been evicted for abusive behaviour. The patient had no family or support network. He had a history of frequent admissions and on one occasion had spent four months in hospital. In the month prior to the specialist social worker's intervention, he was admitted to hospital on three occasions via the Department of Emergency Medicine (DEM). Typically, he would be brought to DEM by the police after being found semi-conscious lying on the ground in a public space. Alternatively, a local homelessness service would organise his transfer to hospital.

Jack has diabetes and is incontinent. He posed many challenges to nursing and allied health staff while in hospital because of his abusive and erratic behaviour. Substitute decision makers were involved because of his cognitive impairments. At the time of his latest admission, DEM staff facilitated a clinical case consultation and he was identified for follow up by the specialist social worker. **Early identification of this potential long stay patient resulted in the specialist social worker calling a stakeholder meeting with executives in DEM and a medical consultant to plan his management, including developing a behavioural management plan**. Identified psychosocial barriers to discharge included homelessness, substitute decision making, lack of family support, challenging behaviours, resistance to being discharged, and finding a suitable residential care facility.

Jack spent a total of ten days in hospital on this occasion. The specialist social worker intervened on day nine and, through intensive case management, was able to negotiate a nursing home placement in a nearby coastal town. Although he was initially uncooperative, the specialist social worker worked with the ward social workers to identify the source of his resistance to discharge. The patient was counselled and encouraged to express his fears about discharge. The specialist social worker used a directive style of communication and negotiated a workable discharge plan for the patient that involved procuring a wheelchair for his use after discharge. He was transferred to the nursing home the next day. While the patient did move into the nursing home, he was later admitted to the local hospital and then self-discharged against medical advice. He subsequently hitchhiked back to the metropolitan hospital where he had been a frequent presenter. However, since that time he has not presented again.

Case 2 - Ben

Ben is a divorced male in his late eighties who had been residing at home at the time of admission. He was from a Filipino background, fluent in English, and had **no family support in Australia.** In the previous year, Ben had five hospital admissions over a nine month period. During the latest admission due to multiple medical conditions and his inability to cope at home, he was admitted to the Geriatric Evaluation and Management (GEM) unit for rehabilitation. While Ben valued his independence, his physical impairments and social isolation put him at risk of further injury. He was assessed as needing residential care because of his inability to carry out activities of daily living.

[1] Note: These case studies are conceptual composite cases highlighting a range of psychosocial barriers to discharge for long stay patients or patients at risk of long stay in hospital. No one case represents a single patient.

Ben presented as angry and resistant to staff's attempts at discharge planning. Although he had signed a residential care agreement, he subsequently displayed an unwillingness to follow through with the residential care pathway. **Despite the ward social worker's attempts to facilitate a placement, three months had elapsed with no progress.** The specialist social worker re-assessed the case and identified the barriers to discharge including a lack of family and social support, a strong resistance to considering residential care options, and financial stress. The specialist social worker worked with the ward social worker and the GEM unit manager to devise a systematic step-by-step case management plan, which included escalating the case to the executive because of the stalemate surrounding the patient's discharge. With executive support, the patient subsequently agreed to enter an interim care facility after his options were put to him in a formal letter. He was re-admitted to hospital after two weeks in interim care, but was later discharged to a nursing home which he attended voluntarily. **Ben's total length of stay was 101 days.**

Case 3 – Mark

Mark is a male in his late seventies and resides in a nursing home. He has a range of cognitive impairments. He was transported to the Department of Emergency Medicine (DEM) at the request of the nursing home. **Despite being assessed as discharge-ready, the nursing home refused to take him back to the facility.** Mark exhibited challenging behaviours including verbally abusive behaviour and inappropriate touching of staff and patients. At admission, the specialist social worker assessed the patient as being at risk of prolonged hospitalisation due to his behavioural issues and the nursing home's failure to comply with its responsibilities. **Although the ward social workers had been advocating for his return to the nursing home, this was met with strong resistance.** As a result of the staff's inability to convince the nursing home to readmit the patient, the specialist social worker became involved with the case two days post-admission. She consulted with the Director of Social Work about strategies to manage the case and subsequently escalated the matter to the hospital executive. The hospital executive, acting on the specialist social worker's advice, reminded the manager that the patient had security of tenure and quoted relevant sections of the Aged Care Act to advocate for his return. The stalemate around discharge was subsequently resolved at the executive level of the hospital and nursing home following a stakeholder meeting initiated by the specialist social worker. **Mark was discharged after six days in hospital.**

Case 4 – Gerry

Gerry is a divorced male in his mid eighties. Prior to his hospital admission he lived independently in his own unit. Gerry was admitted to an acute medical ward after a fall. After 17 days, his condition was classified as non-acute but he subsequently acquired an antibiotic resistant infection and was admitted to the infectious diseases ward. The patient was assessed as needing interim care pending his return home or entering a residential facility. However, his infectious status represented a significant discharge barrier and he was declined admission by one interim care facility. **Forty days post-admission, the specialist social worker intervened.** Through her previous work in establishing a strong working relationship with the interim care facilities in the local area, the specialist social worker was able to advocate for a private room for the patient. **He was discharged from hospital the next day.**

For peer review only

Supplementary File 3: Detailed description of the specialist social worker-led model of care

An innovative specialist social worker-led model of care was developed and piloted in the study site. This social work led model of care took a holistic approach to the management of complex long stay patient discharges and incorporated: micro, patient and family; meso, organisational; and, macro, external strategies and interventions to manage this patient cohort.

Prior to the implementation of this model of care, individual social workers undertook case management responsibility for complex long stay patients as part of their existing caseload within their allocation ward. These social workers had varying levels of experience ranging from newly graduated to experienced; and carried up to 40 cases at any given point. They constantly juggled the demands of immediate discharges with the more specialised requirements of long stay patients. The social workers had little support or guidance beyond monthly clinical supervision sessions to find appropriate solutions to complex discharge barriers, which inherently impacted length of stay. There was ad hoc early identification with no clear escalation processes. There were little to no relationships with key stakeholders and data collection was rudimentary.

In contrast, the new model of care added an additional level of specialised service delivery which was designed to support and enhance the existing social worker service which continued to undertake 'business as usual', but also expedite discharge through a multi-faceted approach. Underpinned by essential elements including the use of a highly skilled specialist worker, a single point of contact and robust data collection and analysis, the model of care also incorporated social work assistant support to maximise efficiency and align tasks to skill level. Internally, the social work department developed processes to ensure the senior social worker was aware of the progress of each long stay case. A social work clinical case management and reporting database called 'Pathfinder' was developed to allow the senior social worker to track, manage, oversee and provide visibility of all patients on the residential aged care, adults with disability and medico-legal pathways. Ward social workers were required to give regular progress updates to the social work assistant who recorded this information in Pathfinder. Once lodged on Pathfinder the senior social assessed all cases, and tailored specific interventions for each case based on the unique psychosocial barriers to discharge. The system also allowed the senior social worker to monitor, track and oversee all cases, as well as providing regular communication to hospital executive through daily reports to hospital executive and other major stakeholders to ensure the organisation had visibility of the progress of these cases.

For peer review only

Another key element to the model of care was the development of clear and timely internal escalation processes to engage hospital executive in decision. Protocols for these interventions were formalised and their benefits were promoted to hospital management and staff to ensure they became embedded into clinicians' everyday practice. The senior social worker, in consultation with the Director of social work escalated stalled cases or 'stranded patients' that posed a significant discharge delay or reputational risk to the organisation due to external institutional delays, family refusal of care, or legal issues.

Externally the model of care implemented pathways and partnerships with key agencies and care providers to ensure timely and appropriate discharges. A nursing home vacancy register was developed alongside a targeted strategy to develop key relationships which resulted in priority notification of vacancies and improved outcomes for patients, especially those with challenging behaviours who previous were difficult to place. Relationships and pathways were also developed with key government agencies, improving their understanding of hospital discharge timeframes and ultimately leading to more responsive turnaround on decisions which had previously taken weeks or months.

An important element of this model was the inclusion of a social work assistant who successfully assisted both the senior social worker and also the ward social workers with sourcing nursing home vacancies using the nursing home vacancy register as well as through the relationships developed with facilities. The role also assisted with time consuming administrative tasks particularly for residential aged care placements such as emails, phone calls, paperwork collation and the maintenance of Pathfinder, nursing home vacancy and other databases. This support was essential to the success of the model by allowing the senior social worker the time to provide the necessary consultations and specialist advice.

Lastly, the model of care also drew on the social workers' specialist skills and knowledge to undertake an advisory role thereby influencing both the organisation and external networks through membership on key committees. The role has been instrumental in the development of new policies and services that ultimately improve outcomes for complex long stay patients. An example of this was the development of strategies for advocating for patients' rights in the face of external institutions' abrogation of their responsibilities to patients such as refusal of ongoing care.

Although developed in a large urban hospital, the model has potential to be modified and scaled to meet local needs and can be implemented without the assistance of a support worker.

For peer review only

CHEERS checklist

Section/item	Item No	Recommendation	Reported on page No/line No
Title and abstract			
Title	1	Identify the study as an economic evaluation or use more specific terms such as "cost-effectiveness analysis", and describe the interventions compared.	Page 1 Line 3
Abstract	2	Provide a structured summary of objectives, perspective, setting, methods (including study design and inputs), results (including base case and uncertainty analyses), and conclusions.	Page 2
Introduction			
Background and objectives	3	Provide an explicit statement of the broader context for the study.<br>Present the study question and its relevance for health policy or practice decisions.	Page 4 paras 1-2<br>Page 4 Para 4
Methods			
Target population and subgroups	4	Describe characteristics of the base case population and subgroups analysed, including why they were chosen.	Page 5 Line 11
Setting and location	5	State relevant aspects of the system(s) in which the decision(s) need(s) to be made.	Page 5 Line 3
Study perspective	6	Describe the perspective of the study and relate this to the costs being evaluated.	Page 5 Line 8
Comparators	7	Describe the interventions or strategies being compared and state why they were chosen.	Page 5 Para 3-4
Time horizon	8	State the time horizon(s) over which costs and consequences are being evaluated and say why appropriate.	Page 5 Line 13
Discount rate	9	Report the choice of discount rate(s) used for costs and outcomes and say why appropriate.	Page 7 Line 10
Choice of health outcomes	10	Describe what outcomes were used as the measure(s) of benefit in the evaluation and their relevance for the type of analysis performed.	Page 6 Line 23
Measurement of effectiveness	11b	*Synthesis-based estimates:* Describe fully the methods used for identification of included studies and synthesis of clinical effectiveness data.	NA
Measurement and valuation of preference based outcomes	12	If applicable, describe the population and methods used to elicit preferences for outcomes.	NA

For peer review only

Section/item	Item No	Recommendation	Reported on page No/line No
Estimating costs and resources	13b	*Model-based economic evaluation:* Describe approaches and data sources used to estimate resource use associated with model health states. Describe primary or secondary research methods for valuing each resource item in terms of its unit cost. Describe any adjustments made to approximate to opportunity costs.	NA
Currency, price date and conversion	14	Report the dates of the estimated resource quantities and unit costs. Describe methods for adjusting estimated unit costs to the year of reported costs if necessary. Describe methods for converting costs into a common currency base and the exchange rate.	Page 7 Line 7
Choice of model	15	Describe and give reasons for the specific type of decision-analytical model used. Providing a figure to show model structure is strongly recommended.	NA
Assumptions	16	Describe all structural or other assumptions underpinning the decision-analytical model.	NA
Analytical methods	17	Describe all analytical methods supporting the evaluation. This could include methods for dealing with skewed, missing, or censored data; extrapolation methods; methods for pooling data; approaches to validate or make adjustments (such as half cycle corrections) to a model; and methods for handling population heterogeneity and uncertainty.	Page 6, line 18 to Page 7, line 16
Results			
Study parameters	18	Report the values, ranges, references, and, if used, probability distributions for all parameters. Report reasons or sources for distributions used to represent uncertainty where appropriate. Providing a table to show the input values is strongly recommended.	Page 7 Line 7
Incremental costs and outcomes	19	For each intervention, report mean values for the main categories of estimated costs and outcomes of interest, as well as mean differences between the comparator groups. If applicable, report incremental cost-effectiveness ratios.	Page 8
Characterising uncertainty	20b	*Model-based economic evaluation:* Describe the effects on the results of uncertainty for all input parameters, and uncertainty related to the structure of the model and assumptions.	NA
Characterising heterogeneity	21	If applicable, report differences in costs, outcomes, or cost-effectiveness that can be explained by variations between subgroups of patients with different baseline characteristics or other observed variability in effects that are not reducible by more information.	Page 8 Line 1 Table 2
Discussion			

For peer review only

Section/item	Item No	Recommendation	Reported on page No/line No
Study findings, limitations, generalisability, and current knowledge	22	Summarise key study findings and describe how they support the conclusions reached. Discuss limitations and the generalisability of the findings and how the findings fit with current knowledge.	Page 9 Line 8 Page 10 Para 3-4
Other			
Source of funding	23	Describe how the study was funded and the role of the funder in the identification, design, conduct, and reporting of the analysis. Describe other non-monetary sources of support.	Page 12 Line 13
Conflicts of interest	24	Describe any potential for conflict of interest of study contributors in accordance with journal policy. In the absence of a journal policy, we recommend authors comply with International Committee of Medical Journal Editors recommendations.	Page 12 Line 11

For peer review only

BMJ Open

A cohort study of a specialist social worker intervention on hospital utilisation for patients at risk of long stay

Journal:	*BMJ Open*
Manuscript ID	bmjopen-2018-023127.R2
Article Type:	Research
Date Submitted by the Author:	08-Oct-2018
Complete List of Authors:	Osborne, Sonya; Queensland University of Technology, Australian Centre for Health Services Innovation<br>Harrison, Gai; Royal Brisbane and Women's Hospital<br>O'Malia, Angela; Royal Brisbane and Women's Hospital, Department of Social Work and Psychology<br>Barnett, Adrian; Queensland University of Technology, Institute of Health and Biomedical Innovation<br>Carter, Hannah; QUT, AusHSI<br>Graves, Nicholas; QUT, IHBI
**Primary Subject Heading**:	Health services research
Secondary Subject Heading:	Health economics
Keywords:	Social work, Long stay patient, Economic evaluation, Cost, Discharge barriers, Length of stay

SCHOLARONE™ Manuscripts

TITLE PAGE

Title

A cohort study of a specialist social worker intervention on hospital utilisation for patients at risk of long stay

Authors

Sonya Osborne | Australian Centre for Health Services Innovation | Queensland University of Technology | Faculty of Health | School of Public Health and Social Work | Kelvin Grove (Brisbane), Queensland 4059 Australia | s.osborne@qut.edu.au

Gai Harrison |Royal Brisbane & Women's Hospital | Department of Social Work |Herston QLD 4029 Australia | gai.harrison@health.qld.gov.au

Angela O'Malia |Royal Brisbane & Women's Hospital | Department of Social Work |Herston QLD 4029 Australia | Angela.OMalia@health.qld.gov.au

Adrian G Barnett | Australian Centre for Health Services Innovation | Queensland University of Technology | Faculty of Health | School of Public Health and Social Work | Kelvin Grove (Brisbane), Queensland 4059 Australia | a.barnett@qut.edu.au

Hannah E Carter* | Australian Centre for Health Services Innovation | Queensland University of Technology | Faculty of Health | School of Public Health and Social Work | Kelvin Grove (Brisbane), Queensland 4059 Australia | hannah.carter@qut.edu.au

Nicholas Graves | Australian Centre for Health Services Innovation | Queensland University of Technology | Faculty of Health | School of Public Health and Social Work | Kelvin Grove (Brisbane), Queensland 4059 Australia | n.graves@qut.edu.au

*Corresponding Author

Word count: 2,879

For peer review only - http://bmjopen.bmj.com/site/about/guidelines.xhtml

ABSTRACT

Background: Long stay patients in acute hospitals commonly present with complex psychosocial needs and utilise high levels of hospital resources.

Objective: To determine whether a specialist social worker-led model of care was associated with a reduction in length of stay for medically stable patients with complex psychosocial needs who were at risk of long stay, and to determine the economic value of this model relative to the decision makers' willingness to pay for bed days released.

Design: A prospective, matched cohort study with historical controls.

Setting: A large, tertiary teaching and referral hospital in metropolitan Southeast Queensland, Australia.

Methods: Length of hospital stay for a cohort of patients seen under the specialist social worker-led model of care was compared with a matched control group of patients admitted to the hospital prior to the introduction of the new model of care using a multistate model with the social worker model of care as an intermediate event. Costs associated with the model of care were calculated and an estimate of the "cost per bed day" was produced.

Results: The model of care reduced mean length of stay by 33 days. This translated to 9,999 bed days released over 12 months. The cost to achieve this was estimated to be AUD $229,000 over 12 months. The cost per bed day released was $23, which is below estimates of hospital decision makers' willingness to pay for a bed day to be released for an alternate use.

Conclusions: The specialist social worker-led model of care was associated with a reduced length of stay at a relatively low cost. This is likely to represent a cost-effective use of hospital resources. The limitations of our historic control cohort selection means that results should be interpreted with caution. Further research is needed to confirm these findings.

For peer review only - http://bmjopen.bmj.com/site/about/guidelines.xhtml

STRENGTHS AND LIMITATIONS OF THIS STUDY

- This is the first study to evaluate a hospital based social work initiative from an economic perspective;
- The small sample size contributed to relatively high levels of uncertainty in the results;
- The use of historical controls who were selected on a different set of criteria to the intervention cohort may have introduced bias or confounding effects beyond those that were controlled for;
- Additional benefits of the model of care, including the potential for improved patient outcomes as well as a reduced workload for existing social workers, were not included in our analysis.

For peer review only

INTRODUCTION

The longer patients remain in hospital after they are medically ready for discharge, the worse the situation becomes for the patient, their families, and for the health system. Patients classified as 'long stay' in acute hospitals are at risk of poor health outcomes and use up valuable hospital resources [1-3]. A retrospective observational study of over 22,000 patients concluded that patients staying in hospital 14 days or longer suffered increased in-hospital morbidity and mortality [4]. Deconditioning and loss of mobility are common outcomes for inpatients who are medically fit for discharge but remain in hospital, with older people being particularly at risk [5]. For patients aged 65 or older, length of stay increased the chance of developing geriatric syndromes in hospital, such as pressure ulcers, incontinence, falls, functional decline and delirium; independent of physical, cognitive or functional impairment [5]. In addition, older people's mobility, muscle strength and aerobic capacity can be adversely affected by just ten days of bed rest, which, alarmingly, translates into almost ten years of functional decline [6]. In recognition of these poor health outcomes, hospitals strive to ensure that patients are discharged in a timely manner once they are deemed medically stable, or fit for discharge, that is, once the medical decision has been made that the patient has completed the required acute care, including all relevant investigations with none further anticipated, and is ready to be discharged from the hospital.

Long stay, that is, the prolonged hospitalisation of patients who are medically fit for discharge, is often the result of non-medical or complex psychosocial barriers to discharge. Patients at risk of long stay commonly present with complex psychosocial needs [7]. Some long stay patients remain in hospital because they lack family support, have limited access to community services, or are waiting to be placed in an interim or long-term care facility [8-10]. Making the unanticipated transition from hospital to institutional care represents a significant life event for many patients and their families, with associated financial and personal costs that may delay discharge. Patients assessed as no longer having capacity to make decisions about their care may similarly face an extended period of hospitalisation, especially if a guardian needs to be appointed to manage their affairs[11]. Material and social disadvantage such as homelessness are also barriers to discharge, while organisational inefficiencies and lack of residential care options may result in placement delays [12 13]. Without timely intervention, patients who present with complex social needs and vulnerabilities are at risk of being stranded in hospital [13].

Typically, long stay patients are referred to hospital social workers, who are spending increasing amounts of time working with this patient cohort to address discharge barriers[9 14-16]. However, there

For peer review only - http://bmjopen.bmj.com/site/about/guidelines.xhtml

is limited evidence on the economic value of hospital social work services, and even less evidence on the economic value of social work initiatives targeting long stay patients.

The purpose of this paper is to report on an evaluation of an innovative specialist social worker-led model of care designed to identify and coordinate timely discharge of long stay patients and those at risk of a long stay in a large teaching hospital. We estimated the cost of delivering the model of care and reported its benefits in terms of bed days released. An assessment of the economic value of the intervention was made using previously reported estimates of Australian hospital decision makers' maximum willingness to pay for a bed day to be released for an alternate use. We used the Consolidated Health Economic Evaluation Reporting Standards (CHEERS) to guide our study report[17].

METHODS

Establishing a Profile of Long Stay Patients

Prior to developing any intervention to address the issue of long stay patients, it was critical to establish a profile of this vulnerable patient cohort. In March 2016, a snapshot one-day audit of several hospital administrative databases was conducted to identify acute patients whose length of stay was equal to or greater than 21 days and sub-acute patients whose length of stay was equal to or greater than 35 days. Patients in intensive care units, maternity wards, mental health wards, and the emergency department were excluded from the audit. A total of 93 patients met the inclusion criteria. Using a purpose-designed audit tool, two persons, a nurse navigator and a senior social worker, independently reviewed each chart to identify barriers to discharge. Eleven psychosocial barriers were identified as risk factors for long stay. These risk factors included family complexity, dysfunction or conflict; domestic and family violence; need for substitute decision making; carer stress; substance abuse; homelessness; challenging patient behaviours; disability; patient or family financial stress; need for residential aged care placement; and disability care planning. These risk factors were in turn able to inform the early identification of patients at risk of long stay. Further explanations of the psychosocial barriers to discharge and several theoretical case study exemplars of the psychosocial complexities of long stay patients can be found Supplementary Files 1 and 2.

Study design, setting and participants

A prospective cohort study with historical controls design was conducted in a large 929 bed, tertiary teaching and referral hospital in metropolitan Southeast Queensland, Australia to evaluate a specialist social worker-led model of care. The primary objective was to determine the association of the specialist social worker-led model of care with lengths of stay for patients who were medically

For peer review only - http://bmjopen.bmj.com/site/about/guidelines.xhtml

stable and fit for discharge before and after introduction of the new model of care. The secondary objective was to determine the economic value of the model of care by estimating the cost per bed day released. We took the perspective of the hospital decision maker and their willingness to pay for a bed day released for an alternate use. The study was approved by the relevant Human Research Ethics Committees for the hospital site (HREC/16/QRBW/476) and the university (QUT1600001102).

The patient cohort included all patients who were entered onto a purpose-built clinical case management database called Pathfinder and managed by the specialist social worker over a three month period from June to August 2016. A historical control group was assembled from hospital administrative databases by selecting a cohort of patients who received usual care before the specialist social worker-led model of care commenced. In order to select a similar group of patients, control patients were those in acute or subacute wards who met the definition of a long stay patient and who had received a social work intervention.

Usual Model of Care (Control)

Prior to the introduction of the new model of care, long stay patients were case managed solely by the allocated ward based social worker who typically held up to 40 cases at any one time of both acute short term and long stay patients. The social work intervention for all cases included psychosocial assessment with view to managing appropriate and timely discharge. Typically referrals for long stay patients were made to social work once the patient was medically stable. There were no early screening processes to identify long stay patients so early intervention was not undertaken. Acute short term patients were often prioritised to maintain rapid patient flow yet there was no framework in place to manage the multiple tasks, meetings, stakeholder liaison, reports and paperwork associated with the care of long stay patients. The service was reactive rather than proactive there was no visibility over delays to social work intervention. Importantly, there was no formal escalation process in place to resolve systemic issues so individual social workers typically attempted to resolve these matters independently, often without success, resulting in lengthy delays.

Specialist Social Worker-led (New) Model of Care (Intervention)

In addition to the individual case management by ward social workers, an additional level of specialised service delivery was added to enhance individual social worker support and expedite discharge through a multi-faceted approach. A process was introduced whereby ward social workers could refer patients they believed to be at risk of long stay to the specialist social worker via clinical consultation. These referrals were subsequently entered on an electronic patient tracking system

For peer review only - http://bmjopen.bmj.com/site/about/guidelines.xhtml

called Pathfinder by the specialist social worker. This allowed the senior social worker to monitor patients at risk of prolonged hospitalisation. The tracking program was a core component of the model of care and was updated daily by ward social workers. As a clinical case management tool, it recorded psychosocial and systemic barriers to discharge as well as current length of stay, which allowed continuous monitoring of patient outcomes. The specialist social worker also regularly collated a list of long stay patients from hospital databases and then consulted with the different social work teams to identify those patients facing psychosocial barriers to discharge. In contrast to the old model of care, the new model was proactive rather than reactive and adopted a systematic approach to identifying and monitoring long stay patients or those at risk of long stay. Key components of the specialist service are listed below, with further detail in Supplementary File 3:

(1) a highly trained and skilled specialist social worker to undertake an overarching advisory role;

(2) a social worker assistant to support the specialist social worker, particularly to manage administrative tasks;

(3) the development of a clinical case management and reporting database called 'Pathfinder' to allow the specialist social worker to design tailored interventions for each patient based on their unique barriers to discharge, and to monitor, track, manage, and oversee all patients on the residential aged care, adults with disability, and medico-legal pathways;

(4) change in communication and reporting processes (e.g. ward social nurses provided regular progress updates to the social worker assistant who recorded the information into Pathfinder; and the specialist social worker regularly communicated through daily reports to hospital executive and other major stakeholders);

(5) development of clear internal escalation processes for 'stranded patients' to engage hospital executives in making timely and appropriate decisions about patient movement and transfer;

(6) implementation of pathways and partnerships with key agencies and care providers to ensure timely and appropriate discharge, including the development of a nursing home vacancy register;

(7) more robust data collection on key performance indicators.

Statistical and Economic Analysis

Characteristics of the specialist social worker-led model of care cohort and the control group were compared using cross-tabulations and summary statistics. Differences between the two groups were tested using the t-test for continuous variables like age, and a chi-squared test for categorical variables like gender. All analyses were made using the R software package (version 3.1.0). The

For peer review only - http://bmjopen.bmj.com/site/about/guidelines.xhtml

difference in length of stay between the patients treated under the new model of care and the control group of patients was estimated using a multistate model.

To estimate the reduction in length of stay we used the models developed by Beyersmann, Allignol, and Schumacher (2011)[18] with the new model of care as an intermediate state between admission and discharge (see Figure 1). Confidence intervals for the length of stay were generated by bootstrapping the data and recalculating the difference in length of stay associated with the social worker intervention. We used 1000 bootstrap estimates.

The bootstrap length of stay estimates were applied in a Monte Carlo simulation to model the cost and length of stay outcomes over a 12 month period. The costs associated with the new model of care are reported in 2017 Australian dollars and include one full-time equivalent specialist social worker for 12 months at a total employment cost of $158,000, plus one full time equivalent social work assistant for 12 months at a total employment cost of $71,000. These costs were assumed to be known with certainty. Discounting of costs was not necessary as the reported time horizon was only 12 months. The activity of the team was reported to be in a range of 20 to 30 patients managed per month, and to model this we used a uniform distribution between 20 and 30. One thousand simulations were made from all distributions to account for the uncertainty in the results. An estimate of the “cost per bed day” was produced to inform judgements around the value for money of the new model of care. This metric recognises that the value of a bed day released may differ according to the decision maker[19]. We used estimates from a recent survey of Australian hospital CEOs which revealed they are willing to pay an average of $216 for a ward bed day released from a quality improving activity[19].

Patient and public involvement:

The study did not involve contact with individual patients. Patients were not involved in setting the research question or the outcome measures, nor were they involved in the design and implementation of the study. Given the vulnerability of the patient cohort we do not plan to disseminate results directly to patients.

RESULTS

A total of 112 patients were included; 52 patients in the specialist social worker led model of care group and 60 patients in the control group. As presented in Table 1, there were some differences between the two groups. The new model of care group was older, with mean difference of 5 years

For peer review only - http://bmjopen.bmj.com/site/about/guidelines.xhtml

(95% CI -1 to 11 years, p-value 0.11). The ratio of men to women was similar in the two groups (Chi-squared test p-value 0.66).

Table 1. Participant Demographics

	Usual model of care (n=60)	Specialist social worker-led model of care (n=52)
**Age, mean (S.D.)**	69 (18)	74 (14)
**Gender, n (%)**		
Female	23(38)	23(44)
Male	37(62)	29(56)

S.D. = standard deviation

The average length of stay for the entire sample was 70 days and ranged from 3 days to 422 days. The median time between admission and involvement of the social worker was 25 days, with an inter-quartile range from 9 to 50 days.

The mean number of bed days expected to be released per patient managed under the new model of care was 33. The distribution around this result was skewed and ranged from 5 to 70 over 1,000 Monte Carlo simulations (Figure 2).

The cost to run the model of care for 12 months was $228,876. Based on the modelled simulations, a total of 9,999 bed days were released over 12 months at an average cost of $23 per bed day released (Table 2).

Table 2 Results of 1,000 Monte Carlo simulations from the input parameter distributions

	Mean	S.D.	Min	Max
Total bed days released per patient managed	33	12	5	70
Average number of patients managed per month	25	3	20	30
Total bed days released per year	9,999	3,781	1140	24,408
Average cost to save one bed day	$23	$16	$9	$201

S.D = standard deviation

When applying the finding that decision makers are willing to pay $216 to release a bed day there was a 100% probability (952/1000 simulations) this model of care will meet the decision criterion. There was an 82% probability (820/1000 simulations) that the cost of saving one bed day was less than $100.

For peer review only - http://bmjopen.bmj.com/site/about/guidelines.xhtml

DISCUSSION

This is the first study to evaluate a hospital based social work initiative from an economic perspective. We found a specialist social worker-led model of care reduced the average length of stay by 33 days, with 9,999 bed days released over 12 months. This translated to a cost of $23 per bed day released. By definition, this study cannot be considered a cost-effectiveness analysis as we did not explicitly account for the value of patients' health outcomes. However, length of stay is now a well-recognised measure of resource use and efficiency in hospitals, with delayed discharge resulting in reduced patient flow and 'access block' for new admissions[20]. Applying the findings of Page et al [19] that hospital decision makers are willing to pay up to $216 to release one bed day, our results indicate there was a 100% chance that adopting the specialist social worker-led model of care described in this study was the right decision, and by extension likely to represent a cost-effective use of resources. This result is supported by the reality that the study hospital has now formally adopted and funded this model of care on a permanent basis.

The new model of care prioritised early identification of patients at risk of long stay, patient tracking, proactive intervention, and system level responses. These four factors promoted a more coordinated and targeted social work response to addressing psychosocial barriers to discharge which in turn assisted in expediting hospital discharge. In addition, early identification of potential long stay patients was facilitated by clinicians' greater awareness of the main psychosocial barriers to discharge when conducting initial psychosocial assessments.

Although the tendency is for hospital social workers to be referred the most complex patients requiring time-intensive interventions[15], hospital-based social workers are under increased pressure to demonstrate that the services they deliver are both effective and cost-efficient[9 14 15 21]. Notably there has been very limited research conducted on the economic value of social work in hospitals. Auerbach, Mason and Hefte LaPorte described the 'cost-containment' value of social work, which they defined as how effective social workers are 'in both serving patients and keeping hospital costs down'[14]. A 2001 study reported on a modelled cost-benefit analysis of social work services in the emergency department (ED) setting, and found that dedicated social work staffing of EDs may yield net economic benefits to a hospital system, especially in large urban centres[22]. However, a literature review did not identify any economic evaluations of hospital based social work initiatives. This may be a reflection of the inter-personal nature of the profession in which outcomes have traditionally been measured subjectively or qualitatively. However, as hospital decision making becomes increasingly focussed on the provision of high value care[23 24], it is important for the social work discipline to demonstrate the economic, as well as clinical, value of their work.

For peer review only - http://bmjopen.bmj.com/site/about/guidelines.xhtml

The comparability of intervention and control groups is a key weakness of our study. Control patients were selected after having met the definition of long stay, while intervention patients were able to be selected without first meeting this definition. This approach enabled us to best reflect the change in practice that occurred given the retrospective nature of our analysis. Specifically, there was no process for the early identification of patients at risk of long stay prior to the intervention, with ward social workers typically becoming involved once patients had met the definition of long stay and were experiencing barriers to discharge. Following the introduction of the new model of care, a focus on early identification meant that patients could be identified, tracked and monitored prior to them meeting the long stay definition. We acknowledge that these differences may have introduced selection bias in the length of stay results. A prospective pre-post study design with clear criteria about eligible patients and a staff member employed to monitor patients and include them would have allowed for the selection of more comparable controls. The sample size of both cohorts was also relatively small which contributed to the uncertainty in cost per bed day results. Not included in this analysis was the potential for the specialist social worker-led model of care to lead to improved patient clinical and quality of life outcomes, as well as a reduced workload for the existing social workers employed by the hospital.

The study was undertaken at a large metropolitan hospital where an oversight role was warranted by the volume of patients being seen. It is unknown whether the efficiencies generated by this role would be replicable in smaller hospital settings. However, the model is scalable with a number of discrete elements that can be adopted separately. There has been interest from other health services in this new model and to date some elements of the model have been adopted by two regional hospitals and one interstate urban hospital.

In conclusion, we found that a specialist social worker-led model of care reduced length of stay in an acute hospital setting at a cost that has been reported as acceptable to hospital decision makers. The model of care is therefore likely to represent a cost-effective use of hospital resources.

For peer review only - http://bmjopen.bmj.com/site/about/guidelines.xhtml

ACKNOWLEDGEMENTS

We acknowledge Sarah Napier, who was instrumental in developing the specialist social worker-led model of care for long stay patients at study hospital, along with Laura Farrelly and Helen Jones who assisted with data collection.

FIGURE LEGENDS

Figure 1: The three states used to measure the change in length of stay due to the social worker intervention

Figure 2: Histogram of the bed days released per patient due to the social worker intervention

DECLARATIONS

Ethics approval and consent to participate

The study was approved by the relevant Human Research Ethics Committees for the hospital (HREC/16/QRBW/476) and the university (QUT1600001102).

Consent for publication

All authors have provided consent for publication of this manuscript.

Competing interests

There are no competing interests to declare.

Funding

Funding in the form of in-kind support was provided under a partnership agreement between the study hospital and the Australian Centre for Health Services Innovation (AusHSI).

Authors' contributions

GH and AO'M made substantial contributions to conception and design of the study; AB, HC, SO provided critical review and contributed feedback on the final protocol and ethics application. AB conceived the statistical analysis plan and conducted statistical analysis and interpretation of data; HC conceived the economic analysis plan; NG contributed to the economic data analysis plan and conducted the evaluation and interpretation of data. SO and HC drafted the final manuscript and critically revised for important intellectual content. All authors read, revised and approved the final manuscript.

For peer review only - http://bmjopen.bmj.com/site/about/guidelines.xhtml

Data Sharing Statement

The full dataset used, which has no identifying information, is available from the corresponding author

For peer review only

For peer review only - http://bmjopen.bmj.com/site/about/guidelines.xhtml

REFERENCES

1. De Coster C, Bruce S, Kozyrskyi A. Use of acute care hospitals by long-stay patients: who, how much, and why? *Canadian Journal of Aging* 2005;24(Suppl 1):97-106.
2. Freitas A, Silva-Costa T, Lopes F, et al. Factors influencing hospital high length of stay outliers. *BMC Health Serv Res* 2012;12:265. doi: 10.1186/1472-6963-12-265
3. Rosman M, Rachminov O, Segal O, et al. Prolonged patients' In-Hospital Waiting Period after discharge eligibility is associated with increased risk of infection, morbidity and mortality: a retrospective cohort analysis. *BMC Health Serv Res* 2015;15:246. doi: 10.1186/s12913-015-0929-6
4. O'Sullivan K, Martensson J, Robbins R, et al. Epidemiology of long-stay patients in a university teaching hospital. *Intern Med J* 2017;47(5):513-21. doi: 10.1111/imj.13379
5. Mecocci P, von Strauss E, Cherubini A, et al. Cognitive impairment is the major risk factor for development of geriatric syndromes during hospitalization: results from the GIFA study. *Dement Geriatr Cogn Disord* 2005;20(4):262-9. doi: 10.1159/000087440
6. Kortebein P, Brock Symons T, Ferrando A, et al. Functional impact of 10 days of bed rest in healthy older adults. *The Journals of Gerontology Series A: Biological Sciences and Medical Sciences* 2008;63(10):1076-81.
7. Jankowski J, Seastrum T, Swidler RN, et al. For lack of a better plan: a framework for ethical, legal, and clinical challenges in complex inpatient discharge planning. *HEC Forum* 2009;21(4):311-26. doi: 10.1007/s10730-009-9117-6
8. Sulman J, Rosenthal CJ, Marshall VW, et al. Elderly Patients in the Acute Care Hospital. *Journal of Gerontological Social Work* 1996;25(3-4):33-52. doi: 10.1300/j083v25n03_04
9. Redfern H, Burton J, Lonne B, et al. Social Work and Complex Care Systems: The Case of People Hospitalised with a Disability. *Australian Social Work* 2015;69(1):27-38. doi: 10.1080/0312407x.2015.1035295
10. Scott IA. Public hospital bed crisis: too few or too misused? . *Australian Health Review* 2010;34(3):317-24.
11. MacKenzie TD, Kukolja T, House R, et al. A discharge panel at Denver Health, focused on complex patients, may have influenced decline in length-of-stay. *Health Aff (Millwood)* 2012;31(8):1786-95. doi: 10.1377/hlthaff.2012.0515
12. Foer D, Ornstein K, Soriano TA, et al. Nonmedical factors associated with prolonged hospital length of stay in an urban homebound population. *J Hosp Med* 2012;7(2):73-8. doi: 10.1002/jhm.992
13. Salonga-Reyes A, Scott IA. Stranded: causes and effects of discharge delays involving non-acute in-patients requiring maintenance care in a tertiary hospital general medicine service. *Aust Health Rev* 2017;41(1):54-62. doi: 10.1071/AH15204
14. Auerbach C, Mason SE, Heft Laporte H. Evidence that supports the value of social work in hospitals. *Soc Work Health Care* 2007;44(4):17-32. doi: 10.1300/J010v44n04_02
15. Galati M, Wong HJ, Morra D, et al. An evidence-based case for the value of social workers in efficient hospital discharge. *Health Care Manag (Frederick)* 2011;30(3):242-6. doi: 10.1097/HCM.0b013e318225e1dd
16. Sims-Gould J, Byrne K, Hicks E, et al. “When Things Are Really Complicated, We Call the Social Worker”: Post-Hip-Fracture Care Transitions for Older People. *Health & Social Work* 2015;40(4):257-65. doi: 10.1093/hsw/hlv069
17. Husereau D, Drummond M, Petrou S, et al. Consolidated Health Economic Evaluation Reporting Standards (CHEERS) statement. *BMJ* 2013;346:f1049. doi: 10.1136/bmj.f1049
18. Beyersmann J, Allignol A, Schumacher M. Competing risks and multistate models with R. New York: Springer 2011.

For peer review only - http://bmjopen.bmj.com/site/about/guidelines.xhtml

19. Page K, Barnett AG, Graves N. What is a hospital bed day worth? A contingent valuation study of hospital Chief Executive Officers. *BMC health services research* 2017;17(1)
20. Qin S, Thompson C, Bogomolov T, et al. Hospital occupancy and discharge strategies: a simulation-based study. *Intern Med J* 2017;47(8):894-99. doi: 10.1111/imj.13485
21. Lechman C, Duder S. Psychosocial severity, length of stay and the role of social work services. *Soc Work Health Care* 2006;43(4):1-13. doi: 10.1300/J010v43n04_01
22. Gordon JA. Cost–Benefit Analysis of Social Work Services in the Emergency Department: A Conceptual Model. *Academic Emergency Medicine* 2001;8(1):54-60.
23. Cassel CK, Guest JA. Choosing wisely: helping physicians and patients make smart decisions about their care. *JAMA* 2012;307(17):1801-2. doi: doi:10.1001/jama.2012.476
24. Owens DK, Qaseem A, Chou R, et al. High-value, cost-conscious health care: concepts for clinicians to evaluate the benefits, harms, and costs of medical interventions. *Annals of internal medicine* 2011;154(3):174-80. doi: 10.7326/0003-4819-154-3-201102010-00007

For peer review only

For peer review only - http://bmjopen.bmj.com/site/about/guidelines.xhtml

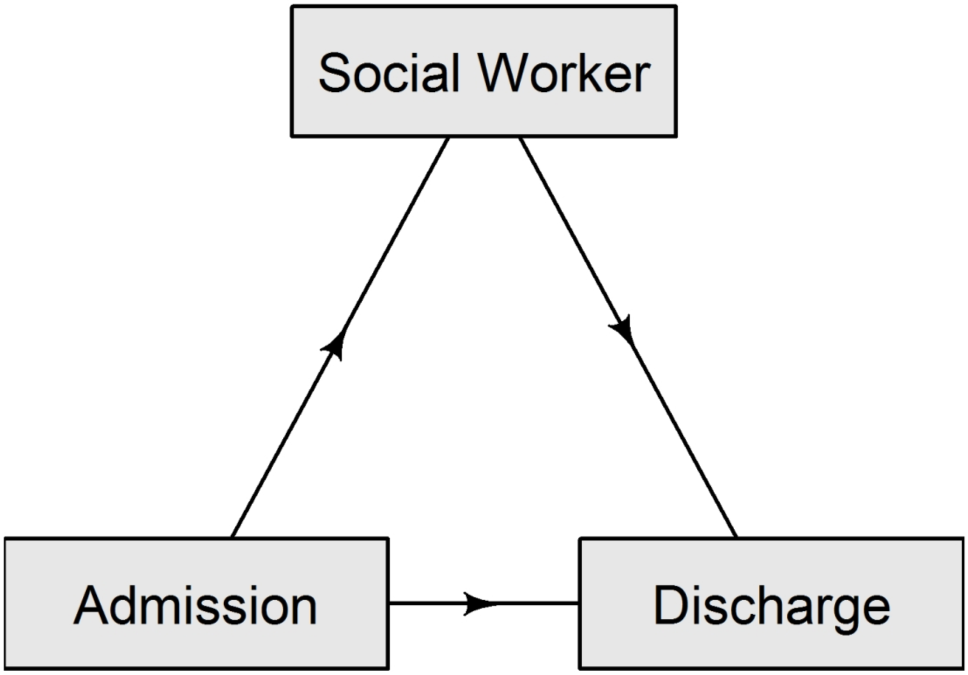

The three states used to measure the change in length of stay due to the social worker intervention

127x101mm (300 x 300 DPI)

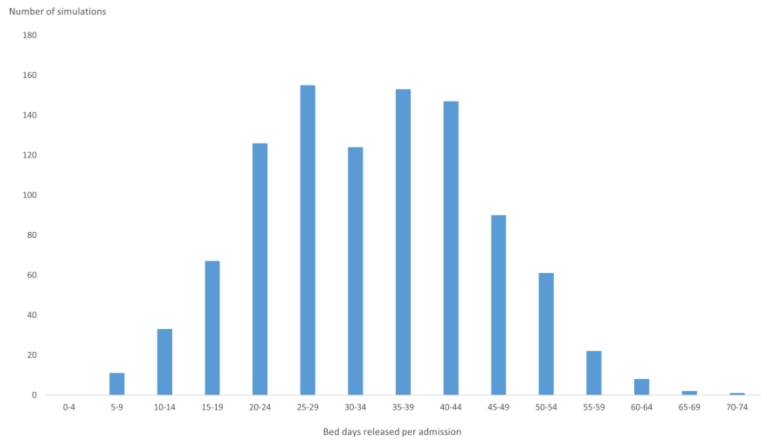

Histogram of the bed days released per patient due to the social worker intervention

338x190mm (300 x 300 DPI)

Supplementary File 1.

Psychosocial Barriers to Discharge Identified from Audit of Long Stay Patients

Barriers to discharge	Examples and/or impact on patient, carer, family
Family complexity (dysfunction or conflict)	Family members may disagree on whether patient should go into residential aged care or go home or disagreeing on care arrangements for a patient post-discharge. This could also include family estrangement, inability to locate family, or situation where the partner of a patient does not want the patient discharged because the partner is currently involved in another relationship.
Domestic and family violence/Elder abuse	This could be suspected or confirmed. It is often difficult to confirm elder abuse, especially if the patient has cognitive impairment, dementia, mental health issues, etc. However, even if abuse cannot be confirmed, the health care professional cannot discount the risk to the patient if he/she is discharged into the care of a suspected abuser.
Substitute decision making	The patient may be awaiting assessment by a civil or administrative tribunal, which is an independent, accessible tribunal that efficiently resolves disputes between parties on a range of matters, such as establishing who is the substitute decision maker if there are complex family dynamics (for example, the patient recently formed a new partnership but has adult children), or the patient has become incapacitated or unable to make sound decisions on their behalf.
Carer stress	Carers indicate that they can no longer manage the demands of caring for the patient and/or are experiencing burnout.
Substance abuse	Patient substance abuse may reduce accommodation options. Substance abuse may also manifest in patient aggression.
Homelessness	For homeless patients, there are usually no viable discharge options.
Challenging behaviours	This is usually in reference to challenging behaviours displayed by the patient, for example, physically or verbally abusive behaviour towards others; not complying with medical care; sexually inappropriate behaviour; dementia related behaviours (e.g. wandering); verbal aggression. These types of behaviours make it harder to find facilities who will accept patients who are likely to impact on the quality of life of other residents or upset staff.
Disability	This usually refers to patients with disabilities where finding suitable out of hospital placements or in-home carers may be difficult.
Financial stress	This could be in relation to the patient and/or the family and could manifest as significant debt or lack of regular income; whereby, patient may not be able to afford residential aged care.
Residential Aged Care	A situation where the patient requires residential care but there is no suitable vacancy or alternatively, the patient may simply be awaiting placement.
Disability Care Planning	A patient may need services at home and be awaiting services at home to be put in place; or there is a lack of suitable accommodation options to meet the disability needs of the patient.

For peer review only

Supplementary File 2. Conceptual Case Study Exemplars[1] of Long Stay Patients with Complex Psychosocial Barriers to Discharge

Case 1 Jack

Jack is an Aboriginal man in his mid-sixties who **at the time of admission had been homeless for several years.** He had previously resided in supported accommodation, but had been evicted for abusive behaviour. The patient had no family or support network. He had a history of frequent admissions and on one occasion had spent four months in hospital. In the month prior to the specialist social worker's intervention, he was admitted to hospital on three occasions via the Department of Emergency Medicine (DEM). Typically, he would be brought to DEM by the police after being found semi-conscious lying on the ground in a public space. Alternatively, a local homelessness service would organise his transfer to hospital.

Jack has diabetes and is incontinent. He posed many challenges to nursing and allied health staff while in hospital because of his abusive and erratic behaviour. Substitute decision makers were involved because of his cognitive impairments. At the time of his latest admission, DEM staff facilitated a clinical case consultation and he was identified for follow up by the specialist social worker. **Early identification of this potential long stay patient resulted in the specialist social worker calling a stakeholder meeting with executives in DEM and a medical consultant to plan his management, including developing a behavioural management plan**. Identified psychosocial barriers to discharge included homelessness, substitute decision making, lack of family support, challenging behaviours, resistance to being discharged, and finding a suitable residential care facility.

Jack spent a total of ten days in hospital on this occasion. The specialist social worker intervened on day nine and, and through intensive case management, was able to negotiate a nursing home placement in a nearby coastal town. Although he was initially uncooperative, the specialist social worker worked with the ward social workers to identify the source of his resistance to discharge. The patient was counselled and encouraged to express his fears about discharge. The specialist social worker used a directive style of communication and negotiated a workable discharge plan for the patient that involved procuring a wheelchair for his use after discharge. He was transferred to the nursing home the next day. While the patient did move into the nursing home, he was later admitted to the local hospital and then self-discharged against medical advice. He subsequently hitchhiked back to the metropolitan hospital where he had been a frequent presenter. However, since that time he has not presented again.

Case 2 - Ben

Ben is a divorced male in his late eighties who had been residing at home at the time of admission. He was from a Filipino background, fluent in English, and had **no family support in Australia.** In the previous year, Ben had five hospital admissions over a nine month period. During the latest admission due to multiple medical conditions and his inability to cope at home, he was admitted to the Geriatric Evaluation and Management (GEM) unit for rehabilitation. While Ben valued his independence, his physical impairments and social isolation put him at risk of further injury. He was assessed as needing residential care because of his inability to carry out activities of daily living.

[1] Note: These case studies are conceptual composite cases highlighting a range of psychosocial barriers to discharge for long stay patients or patients at risk of long stay in hospital. No one case represents a single patient.

For peer review only

Ben presented as angry and resistant to staff's attempts at discharge planning. Although he had signed a residential care agreement, he subsequently displayed an unwillingness to follow through with the residential care pathway. **Despite the ward social worker's attempts to facilitate a placement, three months had elapsed with no progress.** The specialist social worker re-assessed the case and identified the barriers to discharge including a lack of family and social support, a strong resistance to considering residential care options, and financial stress. The specialist social worker worked with the ward social worker and the GEM unit manager to devise a systematic step-by-step case management plan, which included escalating the case to the executive because of the stalemate surrounding the patient's discharge. With executive support, the patient subsequently agreed to enter an interim care facility after his options were put to him in a formal letter. He was re-admitted to hospital after two weeks in interim care, but was later discharged to a nursing home which he attended voluntarily. **Ben's total length of stay was 101 days.**

Case 3 – Mark

Mark is a male in his late seventies and resides in a nursing home. He has a range of cognitive impairments. He was transported to the Department of Emergency Medicine (DEM) at the request of the nursing home. **Despite being assessed as discharge-ready, the nursing home refused to take him back to the facility.** Mark exhibited challenging behaviours including verbally abusive behaviour and inappropriate touching of staff and patients. At admission, the specialist social worker assessed the patient as being at risk of prolonged hospitalisation due to his behavioural issues and the nursing home's failure to comply with its responsibilities. **Although the ward social workers had been advocating for his return to the nursing home, this was met with strong resistance.** As a result of the staff's inability to convince the nursing home to readmit the patient, the specialist social worker became involved with the case two days post-admission. She consulted with the Director of Social Work about strategies to manage the case and subsequently escalated the matter to the hospital executive. The hospital executive, acting on the specialist social worker's advice, reminded the manager that the patient had security of tenure and quoted relevant sections of the Aged Care Act to advocate for his return. The stalemate around discharge was subsequently resolved at the executive level of the hospital and nursing home following a stakeholder meeting initiated by the specialist social worker. **Mark was discharged after six days in hospital.**

Case 4 – Gerry

Gerry is a divorced male in his mid eighties. Prior to his hospital admission he lived independently in his own unit. Gerry was admitted to an acute medical ward after a fall. After 17 days, his condition was classified as non-acute but he subsequently acquired an antibiotic resistant infection and was admitted to the infectious diseases ward. The patient was assessed as needing interim care pending his return home or entering a residential facility. However, his infectious status represented a significant discharge barrier and he was declined admission by one interim care facility. **Forty days post-admission, the specialist social worker intervened.** Through her previous work in establishing a strong working relationship with the interim care facilities in the local area, the specialist social worker was able to advocate for a private room for the patient. **He was discharged from hospital the next day.**

For peer review only

Supplementary File 3: Detailed description of the specialist social worker-led model of care

An innovative specialist social worker-led model of care was developed and piloted in the study site. This social work led model of care took a holistic approach to the management of complex long stay patient discharges and incorporated: micro, patient and family; meso, organisational; and, macro, external strategies and interventions to manage this patient cohort.

Prior to the implementation of this model of care, individual social workers undertook case management responsibility for complex long stay patients as part of their existing caseload within their allocation ward. These social workers had varying levels of experience ranging from newly graduated to experienced; and carried up to 40 cases at any given point. They constantly juggled the demands of immediate discharges with the more specialised requirements of long stay patients. The social workers had little support or guidance beyond monthly clinical supervision sessions to find appropriate solutions to complex discharge barriers, which inherently impacted length of stay. There was ad hoc early identification with no clear escalation processes. There were little to no relationships with key stakeholders and data collection was rudimentary.

In contrast, the new model of care added an additional level of specialised service delivery which was designed to support and enhance the existing social worker service which continued to undertake 'business as usual', but also expedite discharge through a multi-faceted approach. Underpinned by essential elements including the use of a highly skilled specialist worker, a single point of contact and robust data collection and analysis, the model of care also incorporated social work assistant support to maximise efficiency and align tasks to skill level. Internally, the social work department developed processes to ensure the senior social worker was aware of the progress of each long stay case. A social work clinical case management and reporting database called 'Pathfinder' was developed to allow the senior social worker to track, manage, oversee and provide visibility of all patients on the residential aged care, adults with disability and medico-legal pathways. Ward social workers were required to give regular progress updates to the social work assistant who recorded this information in Pathfinder. Once lodged on Pathfinder the senior social assessed all cases, and tailored specific interventions for each case based on the unique psychosocial barriers to discharge. The system also allowed the senior social worker to monitor, track and oversee all cases, as well as providing regular communication to hospital executive through daily reports to hospital executive and other major stakeholders to ensure the organisation had visibility of the progress of these cases.

For peer review only

Another key element to the model of care was the development of clear and timely internal escalation processes to engage hospital executive in decision. Protocols for these interventions were formalised and their benefits were promoted to hospital management and staff to ensure they became embedded into clinicians' everyday practice. The senior social worker, in consultation with the Director of social work escalated stalled cases or 'stranded patients' that posed a significant discharge delay or reputational risk to the organisation due to external institutional delays, family refusal of care, or legal issues.

Externally the model of care implemented pathways and partnerships with key agencies and care providers to ensure timely and appropriate discharges. A nursing home vacancy register was developed alongside a targeted strategy to develop key relationships which resulted in priority notification of vacancies and improved outcomes for patients, especially those with challenging behaviours who previous were difficult to place. Relationships and pathways were also developed with key government agencies, improving their understanding of hospital discharge timeframes and ultimately leading to more responsive turnaround on decisions which had previously taken weeks or months.

An important element of this model was the inclusion of a social work assistant who successfully assisted both the senior social worker and also the ward social workers with sourcing nursing home vacancies using the nursing home vacancy register as well as through the relationships developed with facilities. The role also assisted with time consuming administrative tasks particularly for residential aged care placements such as emails, phone calls, paperwork collation and the maintenance of Pathfinder, nursing home vacancy and other databases. This support was essential to the success of the model by allowing the senior social worker the time to provide the necessary consultations and specialist advice.

Lastly, the model of care also drew on the social workers' specialist skills and knowledge to undertake an advisory role thereby influencing both the organisation and external networks through membership on key committees. The role has been instrumental in the development of new policies and services that ultimately improve outcomes for complex long stay patients. An example of this was the development of strategies for advocating for patients' rights in the face of external institutions' abrogation of their responsibilities to patients such as refusal of ongoing care.

Although developed in a large urban hospital, the model has potential to be modified and scaled to meet local needs and can be implemented without the assistance of a support worker.

For peer review only

CHEERS checklist

Section/item	Item No	Recommendation	Reported on page No/line No
Title and abstract			
Title	1	Identify the study as an economic evaluation or use more specific terms such as "cost-effectiveness analysis", and describe the interventions compared.	Page 1 Line 3
Abstract	2	Provide a structured summary of objectives, perspective, setting, methods (including study design and inputs), results (including base case and uncertainty analyses), and conclusions.	Page 2
Introduction			
Background and objectives	3	Provide an explicit statement of the broader context for the study.<br>Present the study question and its relevance for health policy or practice decisions.	Page 4 paras 1-2<br>Page 4 Para 4
Methods			
Target population and subgroups	4	Describe characteristics of the base case population and subgroups analysed, including why they were chosen.	Page 5 Line 11
Setting and location	5	State relevant aspects of the system(s) in which the decision(s) need(s) to be made.	Page 5 Line 3
Study perspective	6	Describe the perspective of the study and relate this to the costs being evaluated.	Page 5 Line 8
Comparators	7	Describe the interventions or strategies being compared and state why they were chosen.	Page 5 Para 3-4
Time horizon	8	State the time horizon(s) over which costs and consequences are being evaluated and say why appropriate.	Page 5 Line 13
Discount rate	9	Report the choice of discount rate(s) used for costs and outcomes and say why appropriate.	Page 7 Line 10
Choice of health outcomes	10	Describe what outcomes were used as the measure(s) of benefit in the evaluation and their relevance for the type of analysis performed.	Page 6 Line 23
Measurement of effectiveness	11b	*Synthesis-based estimates:* Describe fully the methods used for identification of included studies and synthesis of clinical effectiveness data.	NA
Measurement and valuation of preference based outcomes	12	If applicable, describe the population and methods used to elicit preferences for outcomes.	NA

For peer review only

Section/item	Item No	Recommendation	Reported on page No/line No
Estimating costs and resources	13b	*Model-based economic evaluation:* Describe approaches and data sources used to estimate resource use associated with model health states. Describe primary or secondary research methods for valuing each resource item in terms of its unit cost. Describe any adjustments made to approximate to opportunity costs.	NA
Currency, price date and conversion	14	Report the dates of the estimated resource quantities and unit costs. Describe methods for adjusting estimated unit costs to the year of reported costs if necessary. Describe methods for converting costs into a common currency base and the exchange rate.	Page 7 Line 7
Choice of model	15	Describe and give reasons for the specific type of decision-analytical model used. Providing a figure to show model structure is strongly recommended.	NA
Assumptions	16	Describe all structural or other assumptions underpinning the decision-analytical model.	NA
Analytical methods	17	Describe all analytical methods supporting the evaluation. This could include methods for dealing with skewed, missing, or censored data; extrapolation methods; methods for pooling data; approaches to validate or make adjustments (such as half cycle corrections) to a model; and methods for handling population heterogeneity and uncertainty.	Page 6, line 18 to Page 7, line 16
Results			
Study parameters	18	Report the values, ranges, references, and, if used, probability distributions for all parameters. Report reasons or sources for distributions used to represent uncertainty where appropriate. Providing a table to show the input values is strongly recommended.	Page 7 Line 7
Incremental costs and outcomes	19	For each intervention, report mean values for the main categories of estimated costs and outcomes of interest, as well as mean differences between the comparator groups. If applicable, report incremental cost-effectiveness ratios.	Page 8
Characterising uncertainty	20b	*Model-based economic evaluation:* Describe the effects on the results of uncertainty for all input parameters, and uncertainty related to the structure of the model and assumptions.	NA
Characterising heterogeneity	21	If applicable, report differences in costs, outcomes, or cost-effectiveness that can be explained by variations between subgroups of patients with different baseline characteristics or other observed variability in effects that are not reducible by more information.	Page 8 Line 1 Table 2
Discussion			

For peer review only

Section/item	Item No	Recommendation	Reported on page No/line No
Study findings, limitations, generalisability, and current knowledge	22	Summarise key study findings and describe how they support the conclusions reached. Discuss limitations and the generalisability of the findings and how the findings fit with current knowledge.	Page 9 Line 8 Page 10 Para 3-4
Other			
Source of funding	23	Describe how the study was funded and the role of the funder in the identification, design, conduct, and reporting of the analysis. Describe other non-monetary sources of support.	Page 12 Line 13
Conflicts of interest	24	Describe any potential for conflict of interest of study contributors in accordance with journal policy. In the absence of a journal policy, we recommend authors comply with International Committee of Medical Journal Editors recommendations.	Page 12 Line 11

For peer review only
